# Effect of sleep stage on patterns of fNIRS hemodynamic response to auditory paradigms in 1-month-old Gambian and UK infants

**DOI:** 10.1117/1.NPh.13.S1.S13013

**Published:** 2026-05-25

**Authors:** Maria Rozhko, Merel van der Straaten, Borja Blanco, Isobel Greenhalgh, Johann Benerradi, Sophie E. Moore, Clare E. Elwell, Sarah Lloyd-Fox, Anna Blasi

**Affiliations:** a University of Cambridge, Department of Psychology, Cambridge, United Kingdom; b Wilhelmina Children’s Hospital, University Medical Center, Department of Neonatology, Utrecht, The Netherlands; c King’s College London, Department of Women and Children’s Health, London, United Kingdom; d MRC Unit The Gambia at the London School of Hygiene and Tropical Medicine, Fajara, The Gambia; e University College London, Department of Medical Physics and Biomedical Engineering, London, United Kingdom

**Keywords:** functional near-infrared spectroscopy, brain activation, auditory processing, sleep stages, infants

## Abstract

**Significance:**

The developmental functional near-infrared spectroscopy (fNIRS) literature relies heavily on measurements acquired during natural sleep, yet it remains unclear whether and how different sleep stages modulate infant hemodynamic responses and potentially confound interpretations of early brain activation.

**Aim:**

This study investigates the effect of quiet sleep (QS) and active sleep (AS) on fNIRS-measured hemodynamic responses to two auditory paradigms—social selectivity and habituation and novelty detection (HaND)—in 1-month-old infants from the United Kingdom (N=39) and the Gambia (N=51).

**Approach:**

The infants were tested during natural sleep with an 18-channel bilateral frontal–temporal fNIRS array. Sleep stages were coded from video using a micro-coding scheme.

**Results:**

For the social selectivity paradigm, infants in both cohorts showed robust responses to vocal and non-vocal stimuli and non-vocal selectivity. In the HaND paradigm, UK infants in AS exhibited a higher initial response and stronger habituation in both chromophores than QS infants. In the Gambian cohort, infants in QS showed a more widespread initial response and evidence of habituation, whereas infants in AS did not.

**Conclusions:**

Sleep stage can modulate infant hemodynamic responses, with patterns differing across paradigms and cohorts, underscoring the importance of modeling sleep stages in neuroimaging studies with sleeping infants.

## 1 Introduction

The early postnatal period represents a critical time for studying brain development, marked by intense neural growth and reorganization, including synapse formation and pruning, glial proliferation, and myelination, shaped by both genetic programming and environmental experiences.^1^ Despite the advances in brain imaging technology, acquiring brain imaging data on awake infants in the first weeks of life remains challenging due to limited quiet alertness, frequent movements, and brief visual attention at this age. As a result, infant neuroimaging paradigms often focus on measuring brain responses to auditory stimuli and task-free (resting state) brain activity during natural sleep, building on the evidence from the adult literature that the brain remains receptive to external sensory input even during sleep.^2^ Indeed, investigations conducted in this early postnatal window revealed key insights into the brain’s remarkable capacity for sensory processing and learning. For example, functional near-infrared spectroscopy (fNIRS) studies show that sleeping newborns can discriminate between and remember speech sounds,^3^^,^^4^ recognize speech structure,^5^^,^^6^ phonetic contrasts and prosody,^7^ native versus non-native speech,^8^ and grammatical patterns.^9^^,^^10^ As neuroimaging paradigms are increasingly used to characterize individual differences in brain responses and relate them to later developmental outcomes,^11^ it is essential to show that the evoked response is not simply driven by the participant’s behavioral state (for example, by different sleep stages) or that any state-related effects can be explicitly modeled.

In infant studies, recordings collected during sleep may span one or more cycles of active sleep (AS), a precursor to rapid eye movement (REM) sleep, which is associated with synapse formation and is characterized by increased activity in the sensorimotor region, or quiet sleep (QS), a precursor to non-rapid eye movement (NREM) sleep, which is linked to synaptic pruning and network refinement.^12^ These distinct sleep stages are characterized by distinct patterns of eye and muscle movement, respiration, heart rate, and brain activity.^13^ Given that active and quiet sleep stages serve different functional roles and are associated with distinct neurophysiological profiles, it is important to investigate whether task-evoked brain responses vary across sleep stages in infancy.

Several studies have identified differences in cortical sensory processing depending on the behavioral state of the participant, specifically between sleep and awake states in adults^14^^,^^15^ and infants.^16^^,^^17^ Studies that investigated sleep stage-dependent differences in sensory processing in infants predominantly used electroencephalography (EEG) and magnetoencephalography (MEG).

First, studies have investigated whether the amplitude of neural responses or the proportion of infants showing a significant response differ by sleep stage. These studies have consistently found stronger responses in QS than in AS.^18^^–^^22^ For example, a study of 20 full-term neonates at 2 days of age measured somatosensory evoked potentials using EEG in response to wrist nerve stimulation and found that AS and wakefulness were associated with lower-amplitude neural responses compared with QS.^18^ The same was shown to be true for magnetic fields evoked by auditory speech stimuli, which were also lower in amplitude in AS than in QS, in a group of 10^20^ and 16^21^^,^^22^ newborns. Another study examined somatosensory responses to tactile finger stimulation with MEG in 46 full-term neonates 1 to 23 days of age and found that a significant response was present in 90% of QS infants compared with only 50% of AS infants, indicating a more robust response in QS than in AS.^19^

Second, studies have examined the effect of sleep stage on stimulus discrimination (i.e., the brain’s ability to detect a change in stimulus features). EEG studies on auditory change detection in infants have produced mixed findings, with some suggesting that (a) in neonates, the change detection response is present in AS and QS and the amplitude of response to standard and deviant stimuli does not significantly vary according to sleep stage,^22^^–^^24^ (b) the change detection response is stronger (i.e. there is a larger difference between response amplitude to standard and deviant sounds) in AS compared with QS in neonates^25^ and 2- to 3-month olds^26^ or, on the contrary, (c) the response is stronger in QS compared with AS in 2-month old infants.^27^

Third, a smaller number of studies have explored how sleep stage influences habituation responses, specifically the presence, strength, and timing of neural habituation to repeated stimuli. Based on the evidence of an attenuated and delayed behavioral response to external stimuli in QS compared with AS,^28^ it would be reasonable to expect a faster neural habituation (gradual reduction in response to repeated stimuli) in QS. However, in an EEG study with 9.4±1.2-week-old infants, McNamara et al.^29^ showed that although habituation to tactile stimulation was evident in both AS and QS, AS was associated with a more rapid habituation compared with QS. Similarly, Hunter et al.^30^ examined the auditory sensory gating, using paired clicks (S1→500  ms→S2) presented every 10 s. They averaged S1 and S2 P50 amplitudes across entire NREM/REM periods (72 to 82 trials) to compute S2/S1 ratios (≈1.0 = poor gating) and found robust gating in active/REM sleep but poor gating due to elevated S2 amplitudes in quiet/NREM sleep among 3-month-olds. The effect was stable regardless of the number of averaged trials analyzed.

The hemodynamic response to auditory stimuli, discrimination among auditory stimuli, and habituation have previously been studied using fNIRS in sleeping newborns^5^^,^^31^^–^^38^ and infants aged 2 to 13 months.^17^^,^^39^^–^^42^ Among these, only two studies included sleep stage as a factor, neither of which studied habituation or auditory discrimination. Kotilahti et al.^35^ examined the effect of sleep stage on the location, amplitude, and latency of the peak [defined as the time until the largest change in oxyhemoglobin (HbO) and deoxyhemoglobin (HbR) concentration relative to baseline] of hemodynamic responses to simple sinusoidal beep stimuli in 20 full-term 1- to 3-day-old neonates. Contrary to the results of the EEG and MEG studies, they identified a higher response amplitude in AS compared with QS, but there were no differences in the latency or the cortical location of the response across sleep stages. In a study that investigated response to speech and music sounds in 13 full-term 1- to 4-day-old neonates using fNIRS, no sleep stage effects were found.^36^

### 1.1 Why Is It Important to Examine Sleep Stage Effects?

For stimuli-evoked responses to be informative about individual differences and to be used in investigations of their associations with later developmental outcomes, it is important to establish whether the response of interest is influenced by sleep stage at each developmental period. Identifying differences in sensory processing across sleep stages can clarify the distinct contributions of active and quiet sleep to functional brain development. For example, if infants habituate more readily to repeated stimuli or can discriminate among different stimulus types in one sleep stage compared with another, this would imply the evolutionary role of this sleep stage in early learning that occurs even during sleep. Beyond scientific validity, doing so enables evidence-informed guidance for future work using the same paradigms and ages: if a response varies by sleep stage, studies should invest in the technology, training, and personnel needed to determine sleep stage reliably; if a response proves robust across stages, the paradigm becomes more feasible for clinical applications and data collection outside specialized laboratories, where sleep scoring expertise or equipment may be limited. Consistent with this principle, prior reports recommend restricting analyses to the sleep stage in which the response is most reliable (e.g., using AS for sensory gating^30^ or QS for somatosensory responses^19^) and ensuring groups are matched for the proportion of data acquired in each stage to avoid confounding by behavioral state.

Evidence from EEG and MEG studies suggests that QS is associated with stronger cortical responses to sensory stimuli, whereas AS may support faster or more dynamic processing, such as sensory gating and habituation. The limited evidence from existing fNIRS studies reports a greater hemodynamic response in AS than QS or no clear effect of sleep stage. To our knowledge, to date, no studies have examined how sleep stage influences auditory discrimination or habituation measured with fNIRS in young infants. Taken together, the existing evidence remains mixed and potentially paradigm or imaging modality-dependent, highlighting the need to systematically examine how sleep stage modulates hemodynamic responses at this age. Moreover, all studies discussed so far were conducted in high-income countries, limiting generalizability, because responses to these paradigms, as well as sleep stage effects, may diverge in low- and middle-income settings where social and physical environments, and thus early sensory and sleep experiences, can be substantially different.

### 1.2 Physiological Bases of Sleep Stage Differences in Hemodynamic Responses

Several factors may explain the observed differences in hemodynamic responses between sleep stages. Cerebral blood volume and tissue oxygenation are known to vary across sleep stages, reflecting differences in neural activity and metabolic demand. For example, Doppler flowmetry studies in neonates have shown increased cerebral blood flow during transitions from QS to AS, suggesting elevated metabolic activity associated with AS onset.^43^^,^^44^ Similarly, adult fNIRS studies report elevated oxyhemoglobin concentrations during light sleep and transitions among stages.^45^ However, some neonatal evidence suggests that changes in hemoglobin concentration may not be limited to transition periods: Münger et al.,^46^ using fNIRS in 2- to 8-day-old full-term infants, reported fluctuations in oxyhemoglobin concentrations both during and among transitions from AS to QS and vice versa. Taken together, these findings imply that although some hemodynamic shifts may reflect transitional dynamics, others may be intrinsic to sustained sleep stages themselves.

Supporting this, a longitudinal fNIRS study measuring tissue oxygenation index (TOI), a metric more sensitive to venous saturation and thus oxygen extraction, showed that TOI was lower in AS at 2 to 4 weeks of age, equivalent across stages at 2 to 3 months, and greater in AS by 5 to 6 months.^47^ Given that TOI reflects the balance between cerebral blood flow and oxygen consumption, these findings suggest that AS may initially be characterized by high metabolic demand that outpaces blood flow, leading to greater oxygen extraction. With age, neurovascular regulation matures, and hemodynamic patterns shift to resemble those observed in adults. Thus, the apparent discrepancies across studies may reflect developmental changes in cerebral physiology, rather than true contradictions.

In addition, sleep stages have been shown to be associated with distinct functional connectivity (FC) patterns in neonates; however, again, findings are mixed.^48^^–^^51^ Results of an EEG investigation showed an increase in long-range connectivity in QS, whereas AS was associated with a local increase in occipital connectivity.^48^ To date, there have been two studies of sleep-stage-dependent changes in FC in newborns using fNIRS. The results of both studies found AS to be characterized by more extensive long-range interhemispheric connectivity and QS by stronger short-range connectivity.^49^^,^^50^ A recent functional magnetic resonance imaging (fMRI) investigation of sleep stage effect on functional connectome in the first few weeks of life found no significant difference, although the authors believed their sample to be underpowered and warranted further investigation with a larger sample.^51^

In sum, differences in hemodynamic responses across sleep stages likely reflect both transient and sustained physiological changes, shaped by developmental maturation of neurovascular regulation. Although findings on functional connectivity remain mixed, emerging evidence suggests that QS and AS may support distinct patterns of local and long-range neural communication from early infancy. These physiological and network-level differences raise important questions about how sleep stage might modulate task-evoked brain responses, motivating our investigation of sleep stage effects on response amplitude, stimulus discrimination, and habituation in early development.

### 1.3 Current Study

The present study investigates the influence of infant sleep stages on the hemodynamic responses measured with fNIRS during a battery of standard auditory paradigms. Several neuroimaging methods, including EEG, MEG, fMRI, and fNIRS, are effective for investigating brain function in neonates and infants. Although EEG and MEG provide direct measures of neural activity, fMRI and fNIRS offer indirect measures via hemodynamic responses associated with underlying neural activity. Among these, fNIRS stands out as the most portable, cost-effective, and least susceptible to movement artifacts, making it particularly well-suited for research outside specialized neuroimaging laboratories.^11^ By measuring hemodynamic responses through changes in HbO and HbR signals, fNIRS enables functional characterization of cortical regions.^52^

The data for this study are part of the Brain Imaging for Global Health (BRIGHT) Project,^53^ a prospective longitudinal study examining infant development from term birth to 24 months of age in the United Kingdom and the Gambia. fNIRS data were acquired at each visit at 1, 5, 8, 12, 18, and 24 months of infant age. Only at the 1-month visit was fNIRS acquired during natural sleep, and thus, only the 1-month data are presented here. At both sites, the design included measures of auditory social selectivity, measures of habituation and recovery of response to novelty, and functional connectivity of the resting state. Due to the few infants identified as being in AS compared with QS during functional connectivity analysis (9 AS versus 6 QS in the United Kingdom and 6 AS versus 28 QS in the Gambia), we did not proceed with comparing connectivity patterns between the two sleep stage groups.

Our first objective was to describe infants’ sleep behavior across the session and classify them into active sleep and quiet sleep groups for each paradigm. Second, we aimed to examine how sleep stages modulate hemodynamic responses during two auditory paradigms. Because auditory paradigms such as social selectivity and habituation/novelty detection are commonly administered during sleep in early infancy, it is important to understand how sleep stage may influence the associated neural responses.

For the social selectivity paradigm, we explored whether sleep stages influenced the amplitude or spatial distribution of these responses or the strength of non-vocal over vocal discrimination. Based on prior fNIRS work showing higher response amplitudes in AS than QS regardless of stimulus type,^35^ we hypothesized a generally higher amplitude of hemodynamic response in AS irrespective of condition. However, due to the absence of prior fNIRS studies explicitly testing sleep stage effects on auditory discrimination, we made no directional hypothesis regarding differences in condition discrimination strength among sleep stage groups.

For the habituation and novelty detection (HaND) paradigm, we expected to see a decrease in response amplitude across repeated presentations of the familiar stimulus irrespective of sleep stage in line with previous literature.^54^ We assessed sleep stage differences in response amplitude to repeated auditory stimuli, the spatial distribution of these responses, and the degree of habituation. Although no fNIRS studies to date have directly examined sleep stage effects on cortical habituation, EEG literature suggests stronger habituation may occur in AS relative to QS. Thus, we hypothesized a greater habituation in AS.

In line with the objectives of the BRIGHT Project,^53^ we did not have specific hypotheses regarding the differences between the United Kingdom (UK) and Gambian (GM) cohorts and did not aim to directly compare populations. Rather, the inclusion of two cohorts allowed us to examine whether sleep stage effects on infant hemodynamic responses are consistent across distinct research contexts.

Importantly, the two cohorts were assessed using identical paradigms and closely matched data collection procedures, whereas data were acquired by different study teams in distinct research settings and socio-cultural contexts. Examining the same question across populations is important because socio-cultural, environmental, and biological factors may influence both cognitive processing and physiological responses in infancy. For example, recent fNIRS work examining hemodynamic responses across paradigms in 1-month-old infants in the United Kingdom and the Gambia has shown that context-specific patterns of brain activity are already evident at this age.^55^ Although the inclusion of two populations was not motivated by specific hypotheses about between-population differences, it provides a valuable opportunity to examine the robustness of findings across contexts.

## 2 Materials and Methods

### 2.1 Participants

#### 2.1.1 UK cohort

In the United Kingdom, 62 families were recruited at the Rosie Hospital, Cambridge University Hospitals NHS Foundation Trust at 32 to 36 weeks of gestation, with one family withdrawing before the 1-month time point. Once per week during the recruitment phase, all families visiting for their antenatal visit with a healthy pregnancy were approached and given information about the study. Interested families were followed up via email/phone and recruited into the study. The inclusion criteria included healthy, full-term pregnancy (37 to 42 weeks of gestation), birth weight >2.5  kg, and the absence of any known genetic or neurological conditions.

Demographics from the full UK cohort in the BRIGHT Project can be found in Lloyd-Fox et al.:^53^ in summary, we followed a recruitment strategy that encompassed natural population variance within the region of recruitment. The majority of families lived either in the university town or in surrounding urban or rural communities within a 20-mile radius. A large proportion of the population living in the city of Cambridge and surrounding areas is multi-lingual. Consequently, a significant proportion of our recruited infants were exposed to multiple languages. Families had on average 1.19 (SD 0.4) children (range 1 to 3) including the 1-month-old. Furthermore, around three-quarters of primary caregivers had completed undergraduate education at a university. The study was approved by the National Research Ethics Service East of England Committee, NHS Health Research Authority (REC reference 13/EE/0200), and informed written consent was obtained from all parents before participation.

#### 2.1.2 Gambian cohort

Detailed characteristics of the Gambian site, context, and recruitment strategies have been described in detail previously.^53^ Briefly, the Gambia, located on the west coast of Africa and bordering Senegal, is largely rural, with many families practicing subsistence farming and living in extended households. Despite historically low national income and education levels, school attendance has improved significantly in recent decades. Childcare is typically shared among family members, and Islam plays a central role in shaping family and community life. The Gambian arm of the BRIGHT Project was conducted at the Keneba Field Station of the Medical Research Council the Gambia at the London School of Hygiene and Tropical Medicine in rural West Kiang, a region affected by pronounced seasonal variation in food availability. Participants were recruited from the rural village of Keneba and nearby villages within a 20-km radius. Over the study period, local infrastructure saw notable improvements, such as new roads, enhancing connectivity for the previously more isolated field station. Pregnant women were identified using the West Kiang Demographic Surveillance System and invited to an antenatal clinic visit to assess eligibility if they expressed interest in taking part in the study. The eligibility criteria required women to have a singleton pregnancy, with Mandinka as their primary language, and to be less than 36 weeks pregnant by the time of the first antenatal visit. Infants were included if born at term (37 to 42 weeks of gestation). In total, 222 families were recruited into the study at delivery, with 204 families still enrolled at the time of the 1-month visit (as 8 were stillborn, 7 suffered neonatal death, and 3 withdrew post birth).

Demographics from the full Gambian cohort in the BRIGHT Project can be found in Lloyd-Fox et al.^53^ In summary, families live in multigenerational households with up to 36 members per compound. Polygamy was common within the cohort with 38.8% of fathers having more than one wife. Consequently, although mothers had on average 4.4 children, including the infant enrolled in the study, fathers had on average 6.9, with a range of 1 to 23 children attributed to a single father. For the generation of parents within our cohort, formal schooling was readily available when they themselves were children; therefore, on average, mothers and fathers within the study had completed 3 and 4 years of schooling, respectively. All primary caregivers reported that their first language was Mandinka. In addition, 16.7% of primary caregivers reported that they spoke a second language, and 3.5% of primary caregivers spoke three languages. Ethical approval was granted by the joint Gambia Government—MRC Unit and the Gambia Ethics Committee and the Scientific Coordinating Committee at the MRC Unit The Gambia (SCC 1351). Informed consent was obtained from all parents before participation in writing or via thumbprint.

### 2.2 Sample Size and Power Analysis

The BRIGHT Project was originally designed as a large longitudinal study, and recruitment targets were set at the project level rather than specifically for the present sleep stage analyses.^53^ The available sample for the present analyses was constrained by the number of infants with both usable fNIRS data and reliable sleep stage classification at the 1-month visit. After initial scanning of the videos for assessment of video quality, it became apparent that many videos recorded in the Gambia could not be sleep-stage-classified due to very low lighting used during data collection, which, although optimal for successful fNIRS data collection, obscured most of the classification criteria. This greatly reduced the final sample size. The full fNIRS dataset from this time point was pre-processed and analyzed by Greenhalgh et al.^55^ in a separate investigation into cross-paradigm hemodynamic responses at this age. While that study concentrated on investigating the fNIRS responses in the full sample including all participants from both sites with valid neuroimaging data, the present work includes only the subset with valid sleep stage coding. To comprehensively investigate the effects of sleep stage, we employed two complementary analytic approaches: one based on pre-defined regions of interest (ROIs) identified from the full fNIRS dataset and a second channel-wise analysis to explore potential sleep stage differences in the spatial activation patterns and task-evoked responses.

For the current work, *a priori* power calculations were conducted using the G*Power software (version 3.1) for the main statistical tests planned. For the two-tailed independent-samples t-tests comparing infants in AS and QS, the required total sample sizes at alpha=0.05 and power = 0.95 were 84 infants (42 per group) to detect a large effect (Cohen’s d=0.8), 210 infants (105 per group) to detect a medium effect (d=0.5), and 580 infants (290 per group) to detect a small effect (d=0.2). For the two-tailed one-sample and paired-samples t-tests assessing task-evoked hemodynamic responses within each sleep stage, the corresponding required sample sizes were 23 infants per group for large effects, 54 infants per group for medium effects, and 147 infants per group for small effects, at alpha=0.05 and power = 0.95. The resulting sample sizes for each paradigm and contrast (which differ between the UK and Gambian cohorts and across analyses) are reported in Sec. 3 and in Fig. 2. Given these final numbers, the study is well powered to detect large effects, has limited sensitivity to medium effects, and is unlikely to detect small effects. Accordingly, the findings were interpreted with emphasis on effect sizes and the consistency of patterns across cohorts, rather than on the absence of statistically significant differences alone. To mitigate the constraints imposed by these sample sizes, we additionally implemented permutation-based tests and bootstrap confidence intervals in our ROI- and channel-wise analyses, as detailed in Sec. 2.7.

### 2.3 Experimental Procedure and Stimuli

The BRIGHT study 1-month session comprised a battery of neuroimaging (EEG and fNIRS), behavioral [Neonatal Behavioral Assessment Scale (NBAS), parent–child interaction], and anthropometric measures. The analyses reported in the current work focus on the fNIRS data. The infants were allowed to naturally fall asleep at some point during their visit, usually after a feed and a change of diapers. A researcher would secure the fNIRS headband on the infant’s head before or after the infant fell asleep. During the presentation of the fNIRS paradigms, infants were held by a researcher or parent seated on a comfortable chair. If an infant began to wake, the researcher or parent would attempt to soothe them by gently rocking them back to sleep. Aside from this soothing, they were instructed not to interact with the infant. At both data collection sites, testing room lights were dimmed or switched off to aid infants’ sleep and comfort. Sessions were video-recorded using a camera on a tripod positioned ∼30  cm from the infant, with at least the head and torso in view, to monitor behavior and sleep. The fNIRS recording always followed the same order of auditory paradigms: social selectivity, followed by HaND. The stimuli were presented using a MATLAB^®^ custom-written stimulus presentation framework, Task Engine,^56^ and Psych toolbox on an Apple Macintosh computer. The intensity of the sound presented via Logitech Z130 stereo speakers was adjusted to a measured ≈60  dB at the position of the infant’s head (range across the task: 60.1 to 61.4 dB).

#### 2.3.1 Social selectivity paradigm

The sequence of stimulus presentation has been used in previous research.^57^^–^^59^ Briefly, two types of conditions [vocal (V) and non-vocal (N)] were presented for eight trials each, with a 10- to 12-s silent baseline period in between. The two types of conditions were presented in the same pseudo-random order across infants and were identical across the United Kingdom and the Gambia sites. The vocal condition comprised four non-speech adult vocalizations of two speakers (who coughed, yawned, laughed, and cried). The non-vocal condition comprised four common environmental sounds (running water, toys, bells, and rattles). Both conditions were 8 s long.

#### 2.3.2 Habituation and novelty detection paradigm

The HaND paradigm has been previously described in Lloyd-Fox et al.^60^ Briefly, it consisted of a spoken sentence that was presented repeatedly (UK: “Hi baby! How are you? Are you having fun? Thank you for coming to see us today. We’re very happy to see you;” Gambian: “Denano a be nyadii. I be kongtan-rin? Abaraka bake ela naa kanan njibee bee, n kontanta bake le ke jeh”). Two versions of the sentence were recorded in each language by native English or Mandinka male and female speakers. Each sentence was considered one trial, 8 s long, preceded by a 10-s silent baseline, arranged in a blocked design. The trials were presented in the following order: 15 repetitions of a female speaker, 5 repetitions of a male speaker, and 5 repetitions of the same female speaker. These trials were clustered into the following epochs: familiarization trials 1 to 5 (Fam1), familiarization trials 6 to 10 (Fam2), familiarization trials 11 to 15 (Fam3), novel trials 16 to 20 (novel), and trials 21 to 25 (post-test).

### 2.4 fNIRS Data Acquisition

**Fig. 1:**
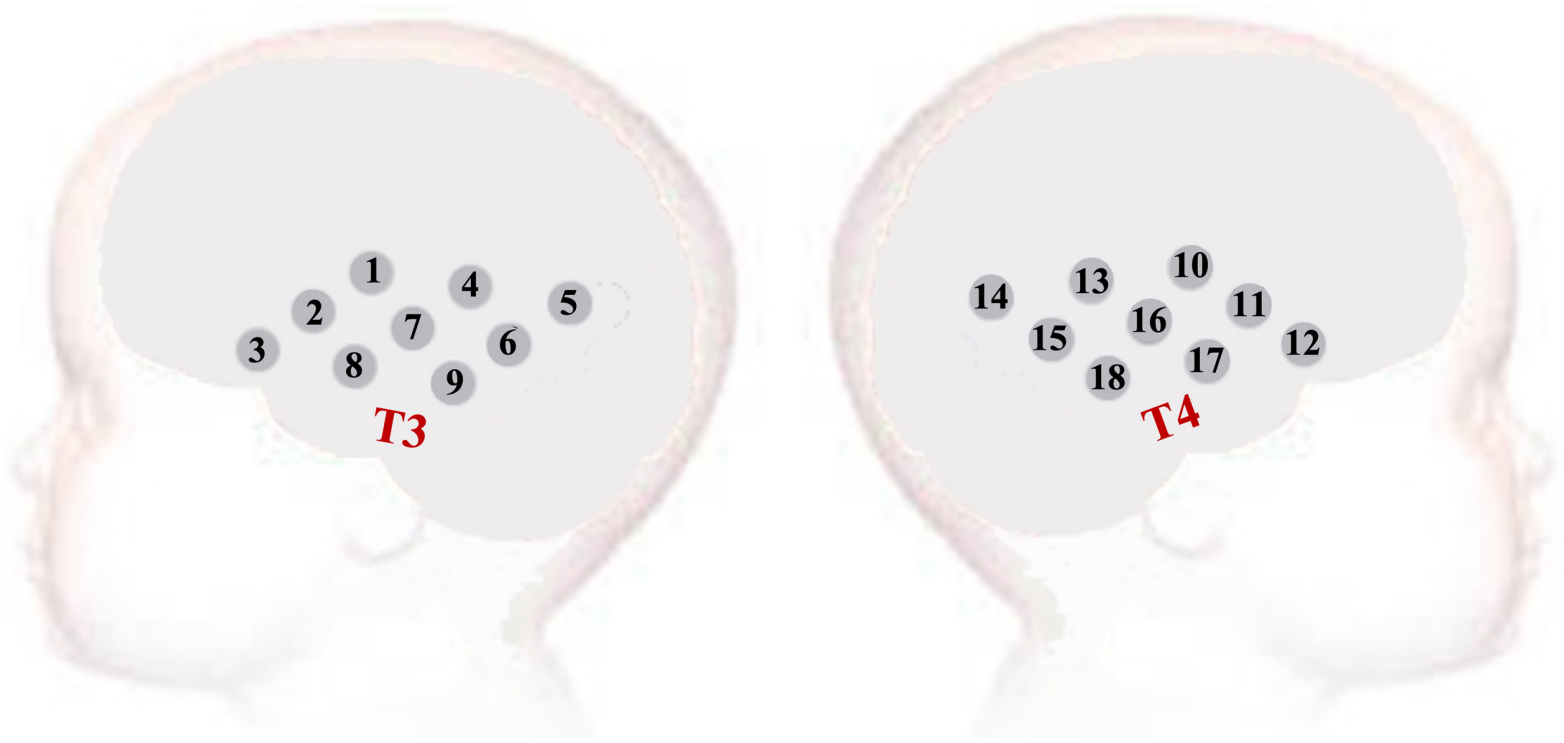
Eighteen-channel fNIRS array used at the 1-month visit, covering the bilateral frontal and temporal regions, with the 10- to 20-system T3 and T4 landmarks clearly marked.

fNIRS data were collected using a continuous wave NTS fNIRS system (Gowerlabs Ltd., London, United Kingdom).^61^ The system makes use of two wavelengths of near-infrared light (780 and 850 nm) to detect changes in HbO and HbR concentrations, using a sampling frequency of 10 Hz. Infants were assessed using a custom-built fNIRS headgear covering both hemispheres and consisting of an array of 8 sources and 8 detectors, for a total of 18 channels (9 per hemisphere), covering bilateral frontal and temporal regions, with a source–detector separation of 2 cm (Fig. 1). For each infant, head measurements were taken to enable the alignment of the headgear with the placement coordinates.^62^ Photographs of the participants were taken after the headbands were secured on the participants’ head and again at the end of the session to facilitate offline coding of the headgear placement and identification of measurement location relative to external anatomical landmarks. With this information, the underlying cortical anatomy of the fNIRS channels was approximated in this study.

### 2.5 fNIRS Preprocessing

The data used in the current analysis were pre-processed by Greenhalgh and colleagues^55^ as part of a separate investigation using the full dataset. Individual channel level quality was assessed using the Quality Testing of Near Infrared Scans (QT-NIRS) function,^63^ with filtering parameters adjusted for infant heart rates (cutoff frequencies: [1.5, 3.5] Hz; scalp coupling index, “SCI”: 0.7; peak spectral power, “PSP”: 0.1  μV). Datasets with more than 40% low-quality channels (based on SCI/PSP thresholds) were excluded. The remaining data were converted from intensity to optical density then underwent motion detection by channel using the Homer2^64^ function (using thresholds: tMask: 1, tMotion: 1 s, STDEVthresh: 15, and AMPthresh: 0.5) and trial rejection if >50% of channels showed motion. Motion correction used spline interpolation (p=0.99) and wavelet filtering (IQR=0.8). Data were then low-pass-filtered (0.6 Hz) and converted to changes in HbO and HbR concentrations using the modified Beer–Lambert law (differential pathlength factors: 5.22, 4.23). Block averaging and linear detrending were applied (tRange for the social selectivity paradigm: −4 to 20 s; for the HaND paradigm: −4 to 18 s). Only datasets with at least three valid trials per condition (i.e., three N and three V trials in the social selectivity paradigm and three Fam1, three Fam3, and three novel trials in the HaND paradigm) were retained. The HbO and HbR values for each channel were extracted for the subsample of infants with valid sleep stage coding and used for the analyses presented here.

### 2.6 Sleep Stage Coding

Although the terms sleep state and sleep stage are often used interchangeably in studies of infant sleep, here we adopt the term sleep stage to refer specifically to AS and QS. This distinction aligns with developmental sleep physiology literature, which classifies AS and QS as distinct stages of infant sleep architecture, each characterized by unique behavioral, cardiovascular, and neural patterns. Throughout this paper, we therefore use sleep stage to denote these two neurophysiologically and functionally discrete phases.

Video recordings of infants during the fNIRS paradigms were reviewed and micro-coded offline to classify periods during which each infant was in AS, QS, or awake. The video was coded in segments of 15 s, following the infant states classification described in the NBAS,^65^ which defines a state as being achieved if the infant remains in that state for at least 15 s. Prior to classifying the infant’s sleep stages, the coders watched 2 min of video recording directly preceding the start of each paradigm to familiarize themselves with the infant’s behavior.

Quiet sleep was scored if the infant’s eyes were closed without rapid eye movements and met at least one additional criterion: regular respiration and no spontaneous activity except (rapid suppression of) startles or movements at regular intervals. Active sleep was scored if the infant’s eyes were closed, though possibly opening briefly, with at least one additional criterion: REMs, low (muscle) activity with random movements and startles or startle equivalents, smoother movements compared with QS, irregular respiration, or intermittent sucking movements. If the infant was not in either sleep stage (e.g., fussy or calmly awake), the state was coded as awake. Each behavioral criterion was assigned a weight (as a percentage of a total possible score of 100%) based on its importance in discriminating sleep stages in newborns, as described in previous literature.^13^ For each 15-s epoch, we summed the weights of all criteria that could be reliably observed (e.g., clearly visible respiration and free limbs allowing movements to be observed), providing an epoch score between 0% (no relevant behavior visible, for example, due to poor lighting) and 100% (all criteria observable). Behaviors that were mutually exclusive among sleep stages (e.g., REM versus non-REM and regular versus irregular respiration) were assigned higher weights than behaviors that could occur in both stages (e.g., startles) (Table S1 in the Supplementary Material). When a criterion could not be coded because of poor visibility, occlusion, or low lighting, its weight was not added to the epoch score. Infants with an average score across all epochs below 65% were excluded from the analyses. Sequentially, infants who remained in one stage throughout the paradigm were allocated to either the AS or QS group.

If an infant transitioned from their initial sleep stage during the paradigm, they were placed in a third ’transition’ group. All trials containing a within-trial transition between quiet sleep and active sleep were excluded to avoid contamination of the hemodynamic response by ongoing stage change. Infants who changed sleep stage during the paradigm were retained if they had at least three valid trials per condition in one continuous block of trials within a single sleep stage. In the social selectivity paradigm, infants were included if they had at least three valid N and three valid V trials in the same sleep stage within a continuous block of six trials; trials from the other sleep stage were discarded. In the HaND paradigm, infants were included in the habituation analysis if they remained in one stage through Fam1, Fam2, and Fam3 and had at least three valid Fam1 and three valid Fam3 trials, and in the novelty-detection analysis if they had at least three valid Fam3 trials directly followed by at least three valid novel trials in the same sleep stage. Each infant contributed data from only one sleep stage per analysis, so that no infant was included in both the AS and QS groups for a given contrast. Within the HaND paradigm, an infant could also contribute data from different sleep stages to different analyses: for instance, an infant might contribute to the Fam1 versus baseline analysis in QS and to the Fam3 versus novelty analysis in AS if the relevant trials met the minimum valid trial criteria in each stage. However, such infants were not included in the habituation analysis (Fam1 versus Fam3) because that contrast required Fam1 and Fam3 trials to come from the same sleep stage. In addition, an infant could contribute data from different sleep stages across paradigms (e.g., QS for the social selectivity task and AS for the HaND task) if they changed state among paradigms and met the minimum valid trial criteria within a single sleep stage for each paradigm separately.

#### 2.6.1 Coders, training, and reliability

Sleep stages were coded from video by three observers. Observer 1 (MR) is a certified NBAS administrator, for whom training and certification include reaching reliability in distinguishing infant behavioral states, including sleep stages, based solely on observation. Because the NBAS manual provides only brief sleep stage descriptions, observers 1 and 2 (MvdS) developed a micro-coding scheme informed by the NBAS, Anders Manual,^13^ and consultation with an experienced infant sleep researcher (Department of Neonatology, Wilhelmina Children’s Hospital, University Medical Centre Utrecht, Utrecht, the Netherlands).^66^ The full coding template and the mapping from these sources are provided in Table S1 in the Supplementary Material. Observers underwent structured training in three steps. First, observer 2 coded three infants jointly with observer 1, discussing the application of each behavioral criterion. Second, observer 2 coded additional infants independently; after every two to three infants, epoch-level reliability with observer 1 was assessed, and discrepancies were reviewed until at least 90% agreement was consistently achieved. Third, observer 2 coded independently, consulting observer 1 only in cases of uncertainty. Observer 3 (RS) was trained by observer 2 using the same staged procedure. Observer 1 contributed to the development of the coding scheme, trained the other observers, and checked reliability on a subset of recordings (20% of datasets coded by observer 2 and 20% coded by observer 3). Observer 2 coded the majority of HaND recordings from the Gambian cohort, and observer 3 coded the social selectivity recordings from both cohorts and additional HaND recordings from the Gambia. This training and monitoring procedure was used to ensure that all observers met a predefined standard of expertise before contributing data to the final analyses.

Two trained coders independently scored sleep stages for 20% of videos (34/170 infants). Inter-rater reliability was excellent for percentage time in quiet sleep (ICC = 0.96) and active sleep (ICC = 0.95) using a two-way random effects model with absolute agreement.

### 2.7 Statistical Analyses

Consistent with prior infant fNIRS research,^58^ we treated a significant increase in HbO and/or a significant decrease in HbR as an indicator of cortical activation. If, however, both chromophores showed significant changes in the same direction—either increasing or decreasing together—this was considered atypical for a functional hemodynamic response and excluded from interpretation.

To compare the quality of the fNIRS data across sleep stages, we computed two metrics: (1) percentage of clean data—defined as the percentage of data out of the total length of the recording (per paradigm) that did not require motion correction—and (2) number of valid trials retained per condition. Independent samples t-tests were used to compare these metrics between the AS and QS groups within each site. Only infants who remained in the same sleep stage throughout the whole paradigm were retained for this analysis.

Two complementary analytical approaches were employed to examine the influence of sleep stage on infants’ hemodynamic responses within the two paradigms. The first approach, referred to as the ROI-based approach, focused on predefined sets of channels that showed significant activation and condition contrasts for each paradigm separately, based on analyses of the full cohort of infants across both sites, as reported by Greenhalgh et al.^55^ Calculation of the ROIs in that work did not account for sleep stage but benefited from the increased statistical power of the larger sample. Greenhalgh et al.^55^ identified significant channels using threshold-free cluster enhancement (TFCE; E=0.5, H=2)—a method that addresses multiple comparison issues and enables detection of significant activation patterns without requiring *a priori* cluster definitions.^67^ The time windows and ROIs derived from the TFCE analysis were then applied to the subsample of infants with valid sleep stage coding to investigate sleep stage-related differences using this ROI-based approach.

The second approach, referred to as the channel-wise analysis, involved an exploratory investigation of sleep stage effects across all channels within the smaller subset of infants for whom sleep stage were reliably coded. This approach enabled assessment of whether hemodynamic responses varied by sleep stage.

All statistical analyses were performed in R (version 2024.04.2+764).^68^ For the primary comparisons of sleep stage effects (ROI-based), distributions of the hemodynamic responses (or difference scores, where applicable) were inspected visually using histograms and Q−Q plots and formally tested for normality (Shapiro–Wilk) within each sleep stage group. Homogeneity of variance for independent sample comparisons was assessed with Levene’s test. Observations with extreme values were identified separately within each sleep stage group using the 1.5×IQR rule (values below Q1−1.5×IQR or above Q3+1.5×IQR) and were excluded from the corresponding t-tests to reduce the undue influence of outliers while preserving the bulk of the data distribution. For all ROI-based comparisons reported in the main text, these assumption checks did not indicate substantial violations, so parametric t-tests were retained.

Given the modest sample sizes and the fact that the present analyses were conducted on a subsample of the original BRIGHT cohort, all ROI-based and channel-wise analyses were complemented by non-parametric resampling procedures. Specifically, for each comparison, permutation tests (5000 label permutations) were used to obtain robust p-values, and Cohen’s d was estimated together with 95% bootstrap confidence intervals (5000 resamples). These permutation-based p-values and bootstrap intervals are reported in the Supplementary Material and led to the same substantive conclusions as the corresponding parametric tests.

#### 2.7.1 Effect of sleep stage on the amplitude of significant activations with the ROI-based approach

The ROI-based approach was first applied to assess the effect of sleep stage on the amplitude of significant activations in the social selectivity and HaND paradigms.

In Greenhalgh et al.,^55^ channels with significant activation to N and V trials compared with baseline were identified separately for the full UK (N=46) and Gambian (N=148) cohorts in the social selectivity paradigm. In that study, both HbO and HbR showed significant responses, with a mixed pattern of HbO and HbR channels across conditions in each cohort (e.g., in the Gambian cohort, 7 HbO and 10 HbR channels showed significant response to N versus baseline, and 5 HbO and 8 HbR channels responded to V versus baseline; in the UK cohort, 9 HbO and 10 HbR channels responded to N versus baseline, and 4 HbO and 2 HbR channels responded to V versus baseline). For the ROI-based analyses examining sleep stage effects on the amplitude of significant activations in the social selectivity paradigm, we defined ROIs by selecting only those channels that showed significant activation to both N and V (versus baseline) within each cohort to maintain an equal number of channels for the two conditions. This resulted in ROIs comprising HbO channels 9, 15, 16, and 18 and HbR channels 15 and 18 for the UK cohort and HbO channels 15, 16, and 18 and HbR channels 6, 7, 9, 10, 13, 15, 16, and 18 for the Gambian cohort. Independent samples t-tests were conducted for each site separately, comparing the AS and QS groups within each social selectivity condition.

To investigate the effect of sleep stage on the amplitude of HbO and HbR responses in the HaND paradigm, we focused on the average responses during Fam1, before considering habituation. Therefore, we used channels identified as having significant activation to Fam1 compared with baseline in Greenhalgh et al.^55^ across the full UK (N=38) and GM (N=136) cohorts. For the UK cohort, Fam1 response was averaged across HbO channels 4 and 7 and HbR channels 7, 15, and 18 in each sleep stage group. For the GM cohort, Fam1 response was averaged across HbO channels 1, 4, 7, and 13 and HbR channels 4, 7, and 13 in each sleep stage group. Independent samples t-tests were conducted for each site separately, comparing the average Fam1 hemodynamic response between the AS and QS groups.

#### 2.7.2 Effect of sleep stage on the strength of the responses for contrasts of interest with the ROI-based approach

Next, the ROI-based approach was applied to assess whether sleep stage influenced the strength of non-vocal selectivity—that is, the degree to which infants responded more strongly to non-vocal than vocal stimuli. The significant regions identified in Greenhalgh et al.^55^ in the full UK cohort for the N > V selectivity were over bilateral posterior temporal regions of the array. Concretely, significant N > V selectivity was present in five HbO channels (channels 4, 13, 14, 15, and 16) and nine HbR channels (channels 4, 7, 9, 13, 14, 15, 16, 17, and 18). There were no channels with significant V > N selectivity. For the Gambian cohort, no N > V or V > N selective regions were observed in the full cohort. Therefore, for the current work, ROI-based analyses of the effect of sleep stage on the strength of N > V selectivity were focused on the UK cohort. After averaging the HbO and HbR responses across the selected channels, condition contrast values were computed for each infant by subtracting the mean V response from the mean N response. These contrast values were then compared between the AS and QS groups using an independent samples two-tailed t-test.

Similarly, the ROI-based approach was applied to assess whether sleep stage influenced the degree of habituation in the HaND paradigm. The regions with significant habituation (Fam1 > Fam3) identified in Greenhalgh et al.^55^ in the full UK cohort were observed over the posterior parts of the array. Significant habituation was present in two HbO channels (channels 4 and 7) and one HbR channel (channel 7). A response to novel stimulus after the habituation phase (novel > Fam3) was not present. For the Gambian cohort, the significant regions were observed in more central temporal regions of the array. Significant habituation was present in two HbO channels (channels 1 and 7) and three HbR channels (channels 4, 7, and 13). As with the UK cohort, no novelty response at the group level was observed. Therefore, ROI-based investigation of the sleep stage effect focused on the Fam1 > Fam3 response in both cohorts. We computed the contrast value for each infant by subtracting the mean Fam3 response from the mean Fam1 response. These contrast values were then compared between the AS and QS groups using an independent sample t-test.

#### 2.7.3 Effect of sleep stage on the fNIRS responses with the channel-wise approach

For the channel-wise analyses, the hemodynamic responses within the QS and AS infants were assessed separately. First, we identified the channels with significant activation to each condition compared with baseline using two-tailed one-sample t-tests, with Benjamini–Hochberg^69^ correction applied across the 18 channels to control the false discovery rate (FDR) (α=0.05). Next, the channels that showed a significant response to condition (N > baseline, Fam1 > baseline, and novel > baseline) were examined for selectivity to paradigm conditions (N versus V in the social selectivity paradigm or Fam1 versus Fam3 and novel versus Fam3 for the HaND paradigm), using two-tailed paired-sample t-tests, with Benjamini–Hochberg FDR correction applied across the 18 channels.

## 3 Results

### 3.1 Characteristics of the UK and Gambian Cohorts

Only participants who contributed valid fNIRS and sleep stage data were retained for the current analysis. The reasons for participant retention and exclusion are outlined below.

#### 3.1.1 UK cohort

**Fig. 2:**
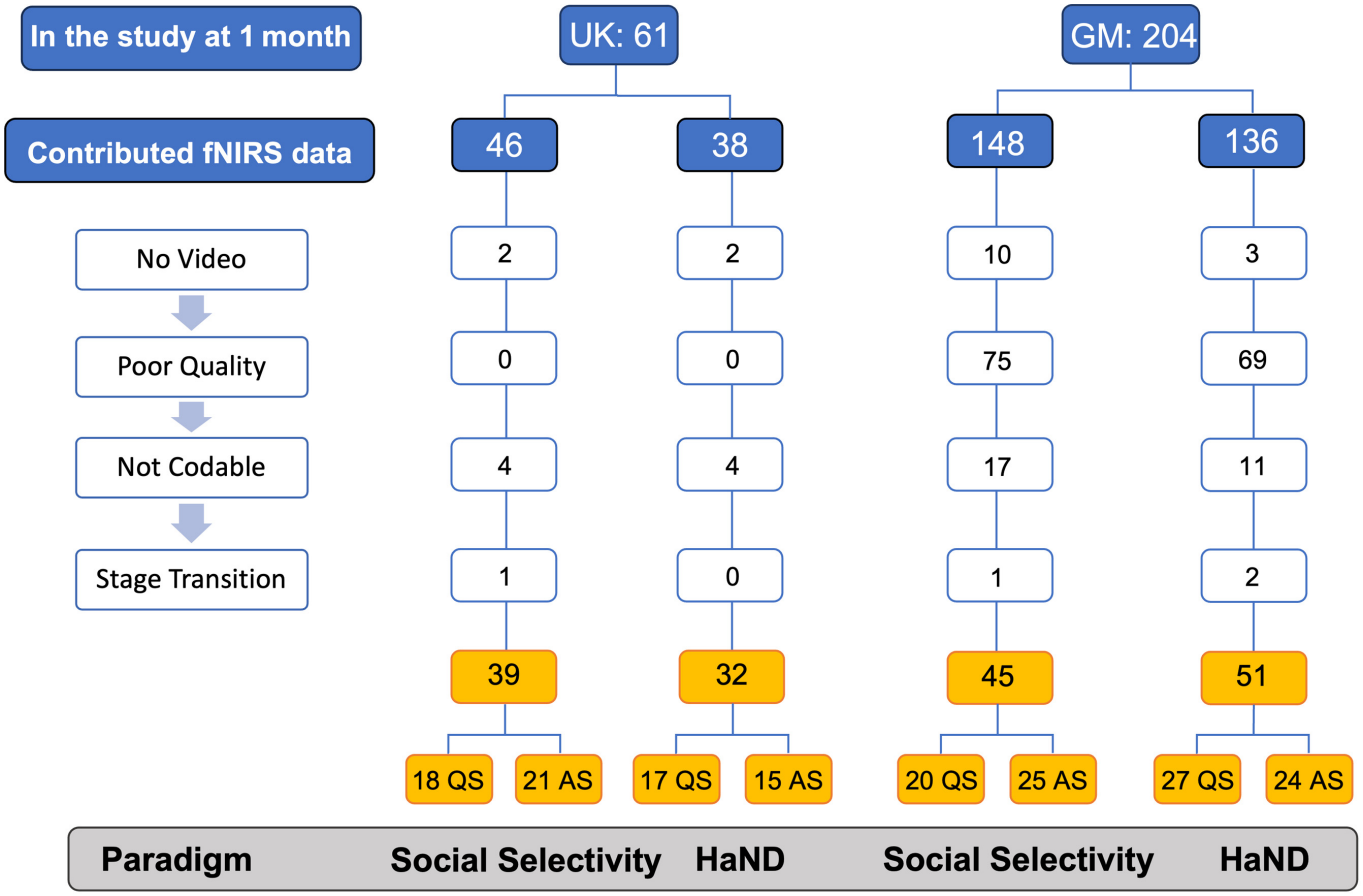
Reasons for exclusion of participants from the UK and GM cohorts from the final analysis across the two paradigms: social selectivity and HaND. The reasons for exclusion could be as follows: no video—video of the whole session or the specific task is missing; poor quality—behaviors cannot be coded because of insufficient lighting in the testing room; not codable—behaviors cannot be coded from the video because infant is facing away from the camera and is wrapped up or in a position that makes coding behaviors not possible; and stage transition—infant transitioned from one sleep stage to another during the paradigm and there are not enough trials of each condition (for social selectivity and HaND) in either stage.

Of the total participants eligible at 1 month (N=61), 46 had valid fNIRS data for the social selectivity paradigm and 38 for the HaND paradigm (Fig. S1 in the Supplementary Material). During the social selectivity paradigm, out of 40 infants with valid sleep stage classification (Fig. 2), 8 infants (20%) transitioned between sleep stages (4 infants changed stage from QS to AS; 2 infants, from AS to QS; and 1 infant changed between AS and awake three times and was excluded from further analysis), 14 infants (35%) remained in QS, and 18 infants (45%) remained in AS during the entire paradigm. After incorporating valid trials from infants that changed sleep stage, the final sample comprised 18 QS and 21 AS infants. During the habituation paradigm, out of 32 infants with valid sleep stage classification (Fig. 2), 3 infants (9%) transitioned between sleep stages (1 infant changed stage from AS to awake, 1 infant from QS to AS, and 1 infant from QS to AS), 13 infants (41%) remained in AS, and 16 infants (50%) remained in QS throughout the paradigm. All three infants that changed stage were retained for further analysis, bringing the total sample size to 32 infants (17 QS and 15 AS) for the analysis of Fam1 response, 16 QS and 15 AS infants contributed data for the habituation (Fam1 to Fam3) analysis, and 16 QS and 14 AS were retained for novelty detection (Fam3 to Novel) analysis. The number, age, and sex of participants who contributed data for the analysis are presented in Table 1.

**Table 1:** Number of UK and GM infants, their mean age (days), and sex (M, male; F, female) that contributed fNIRS and sleep stage data for each paradigm (SD in parentheses).

Site	Paradigm	Sleep stage	N	Mean age (SD), days	Sex
United Kingdom	Social selectivity	QS	18	31.6 (5.2)	8 F, 10 M
		AS	21	34.3 (6.7)	10 F, 12 M
	HaND (Fam1 to Fam3)	QS	16	33.7 (7.3)	6 F, 10 M
		AS	15	32.4 (5.4)	10 F, 5 M
Gambia	Social selectivity	QS	20	35.1 (4.9)	10 F, 10 M
		AS	25	37.0 (6.9)	13 F, 12 M
	HaND (Fam1 to Fam3)	QS	25	36.4 (6.0)	14 F, 11 M
		AS	20	37.1 (6.0)	11 F, 9 M

#### 3.1.2 Gambian cohort

Of the total number of participants eligible at 1 month (N=204), 148 had valid fNIRS data for the social selectivity paradigm, and 136 had valid fNIRS data for at least the habituation part (Fam1, Fam2, and Fam3) of the HaND paradigm. Participant retention per paradigm based on fNIRS data quality in the two cohorts is presented in Fig. S1 in the Supplementary Material. For the social selectivity paradigm, a total of 46 infants contributed both valid fNIRS and sleep stage data (Fig. 2). Eight infants (17%) transitioned between sleep stages (3 infants changes stage from QS to AS, 4 infants from AS to QS, and 1 infant had multiple transitions between AS and awake state and was excluded from further analysis), 16 infants (35%) remained in QS, and 22 infants (48%) remained in AS during the entire paradigm. After incorporating the trials from the infants that changed sleep stage, the resulting sample included 20 QS and 25 AS infants. For the HaND paradigm, a total of 52 infants contributed both fNIRS and sleep stage data (Fig. 2). Eleven infants (21%) transitioned between sleep stages (8 infants transitioned from AS to QS, 2 infants from QS to AS, and 2 infants had 2 changed: AS – QS – AS and QS – AS – QS), 25 infants (48%) remained in QS, and 16 infants (31%) remained in AS throughout the entire task (Fam1 – novel trials). After incorporating the data from infants who transitioned between sleep stages, there were 27 QS and 24 AS infants that contributed data for the analysis of Fam1 response, 25 QS and 20 AS infants that contributed the data for the habituation (Fam1 to Fam3) analysis, and 29 QS and 18 AS that contributed the data for the novelty detection analysis (Fam3 to novel). The number, age, and sex of participants who contributed data for the analysis are presented in Table 1.

### 3.2 Data Quality According to Sleep Stage

Data quality for both paradigms and in both sleep stage groups was greater than 95% (Table 2), and statistical comparison tests showed that for AS, the data quality was significantly lower compared with QS. The number of valid trials retained for analysis was also significantly lower in AS compared with QS for both social selectivity paradigm conditions (N and V) in both cohorts, except for the number of valid V trials in the United Kingdom. For the HaND paradigm, significantly more valid Fam1 and Fam3 trials were retained for QS compared with AS infants in the GM cohort. In the UK cohort, a significant difference was found only for Fam3 trials with more valid trials in the QS group than in the AS group.

**Table 2:** Data quality comparison according to sleep stage for each paradigm in the UK and GM cohorts.

Paradigm	Data quality criteria	Site	Active sleep mean (SD)	Quiet sleep mean (SD)	Test statistic (t)	Significance	Cohen’s d	95% Bootstrap CI (d)	Permutation p
Social selectivity	Percentage clean data, %	United Kingdom	95.3 (3.0)	99.9 (0.3)	t=−6.5	<0.001***	−2.04	[−3.29, −1.43]	<0.001***
		Gambia	95.0 (3.2)	99.3 (1.4)	t=−5.6	<0.001***	−1.66	[−2.53, −1.13]	<0.001***
	Number of valid N trials	United Kingdom	6.1 (1.4)	7.7 (1.1)	t=−3.9	<0.001***	−1.34	[−2.65, −0.58]	0.001***
		Gambia	6.5 (0.9)	7.8 (0.6)	t=−5.2	<0.01**	−1.58	[−2.50, −1.01]	<0.001***
	Number of valid V trials	United Kingdom	6.8 (0.8)	7.5 (1.3)	t=−1.7	0.10	−0.65	[−2.33, 0.09]	0.10
		Gambia	6.6 (1.4)	7.6 (0.7)	t=−2.7	0.01*	−0.81	[−1.39, −0.31]	0.01*
Habituation and novelty detection	Percentage clean data, %	United Kingdom	97.8 (2.6)	99.7 (0.5)	t=−2.7	0.02*	−1.11	[−1.83, −0.60]	0.005**
		Gambia	96.4 (3.0)	98.7 (1.9)	t=−2.8	0.01**	−0.95	[−1.83, −0.31]	0.01**
	Number of valid Fam1 trials	United Kingdom	4.9 (0.4)	5.0 (0.0)	t=−1.5	0.17	−0.62	[−1.14, −0.42]	0.20
		Gambia	4.3 (0.8)	4.8 (0.5)	t=−2.4	0.03*	−0.81	[−1.65, −0.18]	0.03*
	Number of valid Fam3 trials	United Kingdom	4.3 (0.9)	4.9 (0.3)	t=−2.3	0.04*	−0.96	[−1.81, −0.29]	0.04*
		Gambia	4.5 (0.9)	4.7 (0.5)	t=−1.1	0.30	−0.38	[−0.97, 0.32]	0.38
	Number of valid novel trials	United Kingdom	4.6 (0.7)	4.9 (0.3)	t=−1.3	0.21	−0.52	[−1.21, 0.23]	0.27
		Gambia	4.1 (1.3)	4.7 (0.5)	t=−1.8	0.08	−0.67	[−0.03, 1.27]	0.09

Asterisks indicate significance levels: *p<0.05, **p<0.01, ***p<0.001.

### 3.3 Results of the ROI-Based Analyses

#### 3.3.1 Sleep stage effect on the amplitude of the hemodynamic response

**Fig. 3:**
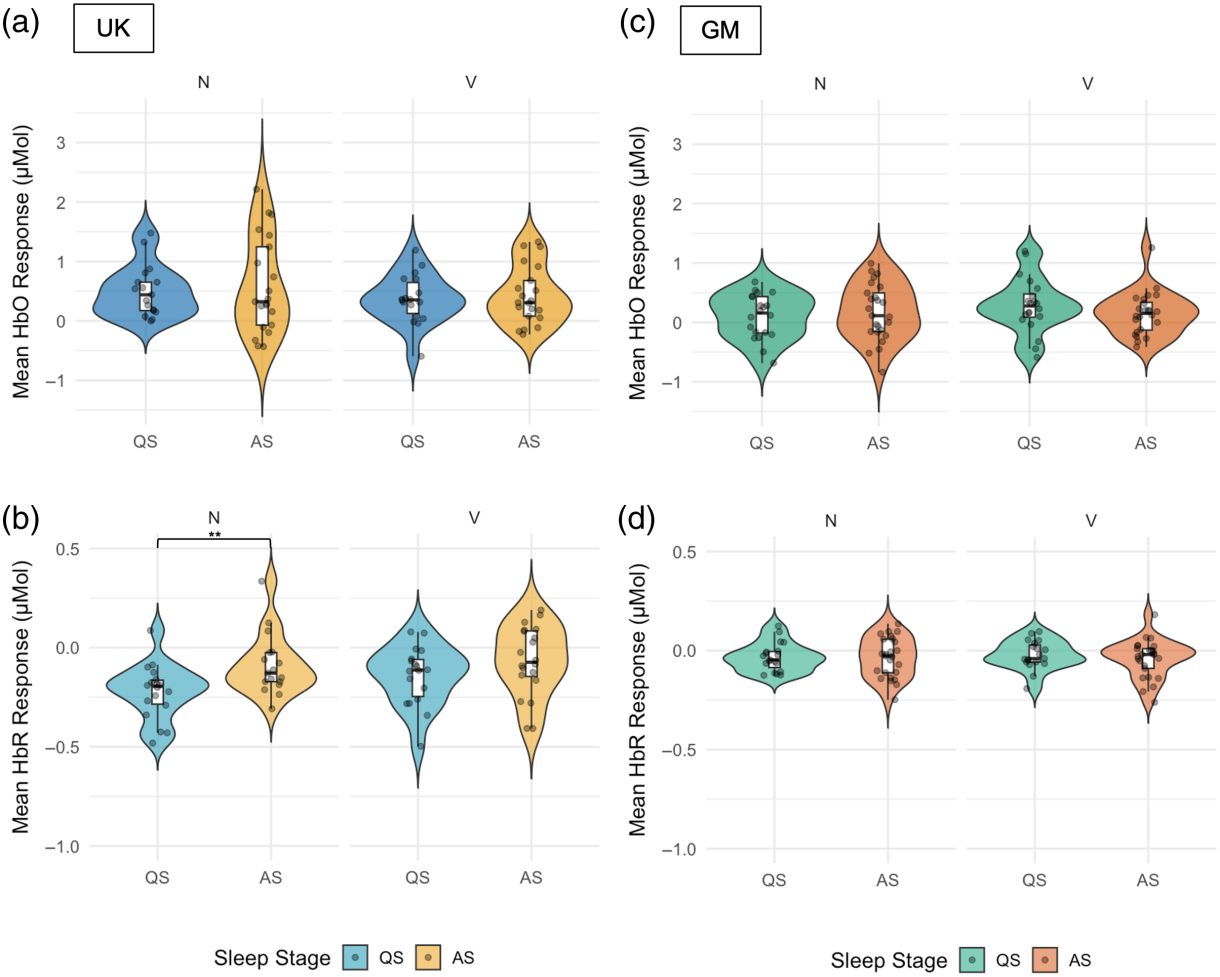
Distribution of mean HbO [(a) and (c)] and HbR [(b) and (d)] response amplitudes (μMol) to non-vocal (N) and vocal (V) trials of the social selectivity paradigm across sleep stages (QS, quiet sleep; AS, active sleep) in the UK [(a) and (b)] and GM [(c) and (d)] cohorts. Each plot illustrates individual data points, overall distributions, and embedded boxplots. Asterisks indicate significance levels: *p<0.05, **p<0.01, ***p<0.001.

We compared the mean amplitude of the hemodynamic response to vocal and non-vocal conditions of the social selectivity paradigm and to the first familiarization epoch (Fam1) of the HaND paradigm between sleep stage groups. For the social selectivity paradigm, the effect of sleep stage was present only for the non-vocal (N) condition and only in the HbR signal in the UK cohort, with a significantly larger (i.e., more negative) response in QS compared with AS [QS M=−0.22  μMol, SD=0.14; AS M=−0.14  μMol, SD=0.15, t(32)=−2.75, p=0.01; Cohen’s d=−0.93 (large)] (Fig. 3). In the GM cohort, the mean HbO or HbR response amplitude did not differ by sleep stage for either condition (see Table S2 in the Supplementary Material for full statistics). Permutation-based p-values and bootstrap confidence intervals for Cohen’s d are reported in Table S2 in the Supplementary Material and led to the same conclusion.

**Fig. 4:**
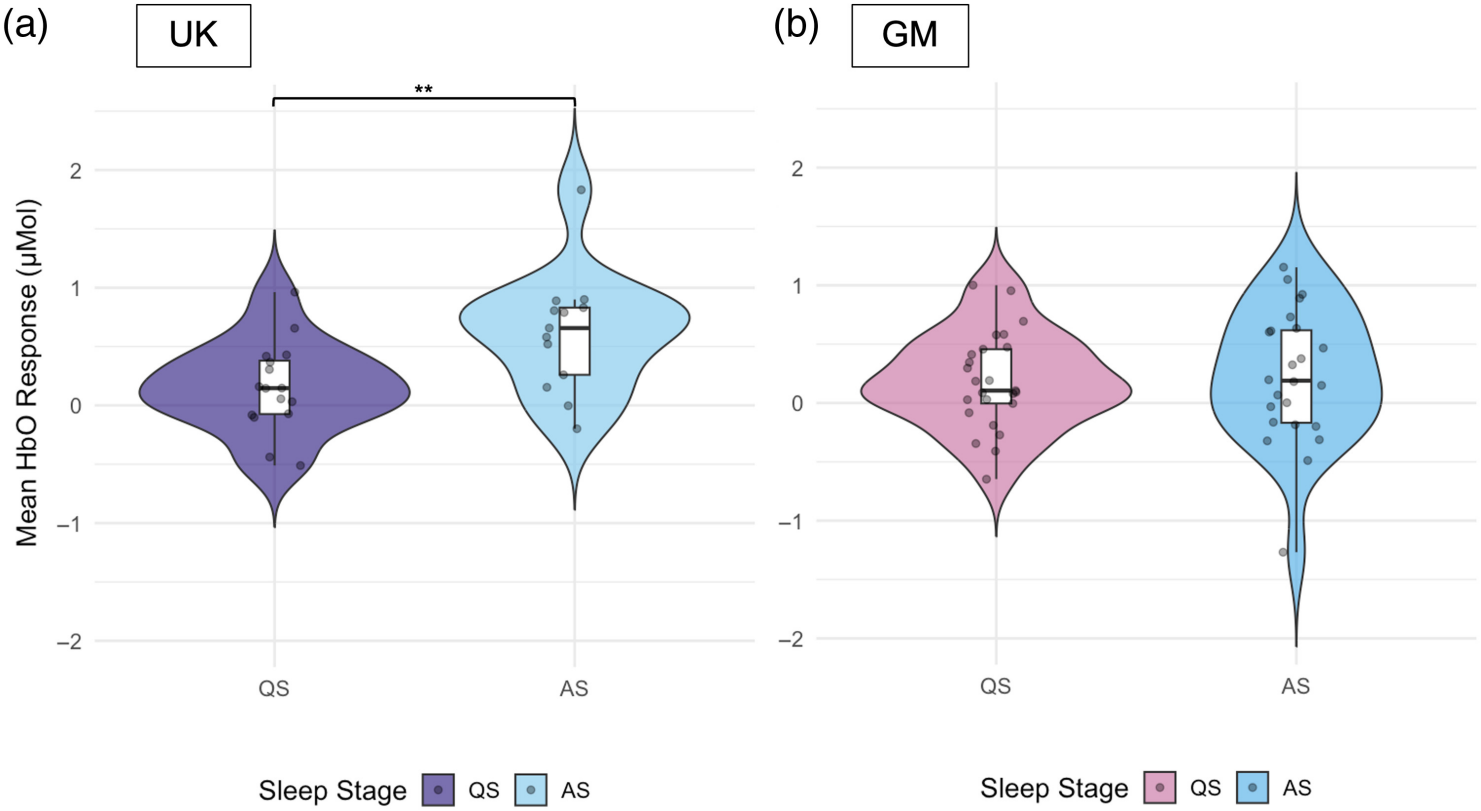
Distribution of the mean HbO response amplitudes (μMol) to the first five familiarization trials (Fam1) of the HaND paradigm across sleep stages (QS, quiet sleep; AS, active sleep) in the UK (a) and GM (b) cohorts. Asterisks indicate significance levels: *p<0.05, **p<0.01, ***p<0.001.

For the HaND paradigm in the UK cohort, the HbO responses during Fam1 were found to be significantly higher in AS compared with QS [QS M=0.15  μMol, SD=0.37; AS M=0.62  μMol, SD=0.51, t(21)=−2.72, p=0.01; Cohen’s d=−1.05 (large)] (Fig. 4). Permutation-based p-values and bootstrap confidence intervals for Cohen’s d are reported in Table S3 in the Supplementary Material and led to the same conclusion. The effect was not present in HbR (p=0.51; Fig. S2 in the Supplementary Material). In the GM cohort, we did not observe an effect of sleep stage on the amplitude of HbO or HbR responses during Fam1 (all p>0.80; see Fig. 4 and Fig. S2 in the Supplementary Material). See also Table S3 in the Supplementary Material for full test statistics, permutation p-values, and bootstrap confidence intervals.

#### 3.3.2 Effect of sleep stage on the strength of non-vocal selectivity

A two-sample two-tailed t-test comparing average N – V condition contrast values among sleep stage groups revealed no significant difference for HbO (QS M=0.30  μMol, SD=0.52; AS M=0.31  μMol, SD=0.60, t(34)=0.08, p=0.93, permutation p=0.95, bootstrap Cohen’s d=0.03 (negligible), 95% CI [−0.66,0.71]) or HbR (QS M=−0.11  μMol, SD=0.13; AS M=−0.09  μMol, SD=0.19, t(36)=0.28, p=0.78, permutation p=0.78, Cohen’s d=0.09 (negligible), 95% CI [−0.54,0.75]) in the UK cohort (Fig. S3 in the Supplementary Material).

#### 3.3.3 Effect of sleep stage on the habituation strength

**Fig. 5:**
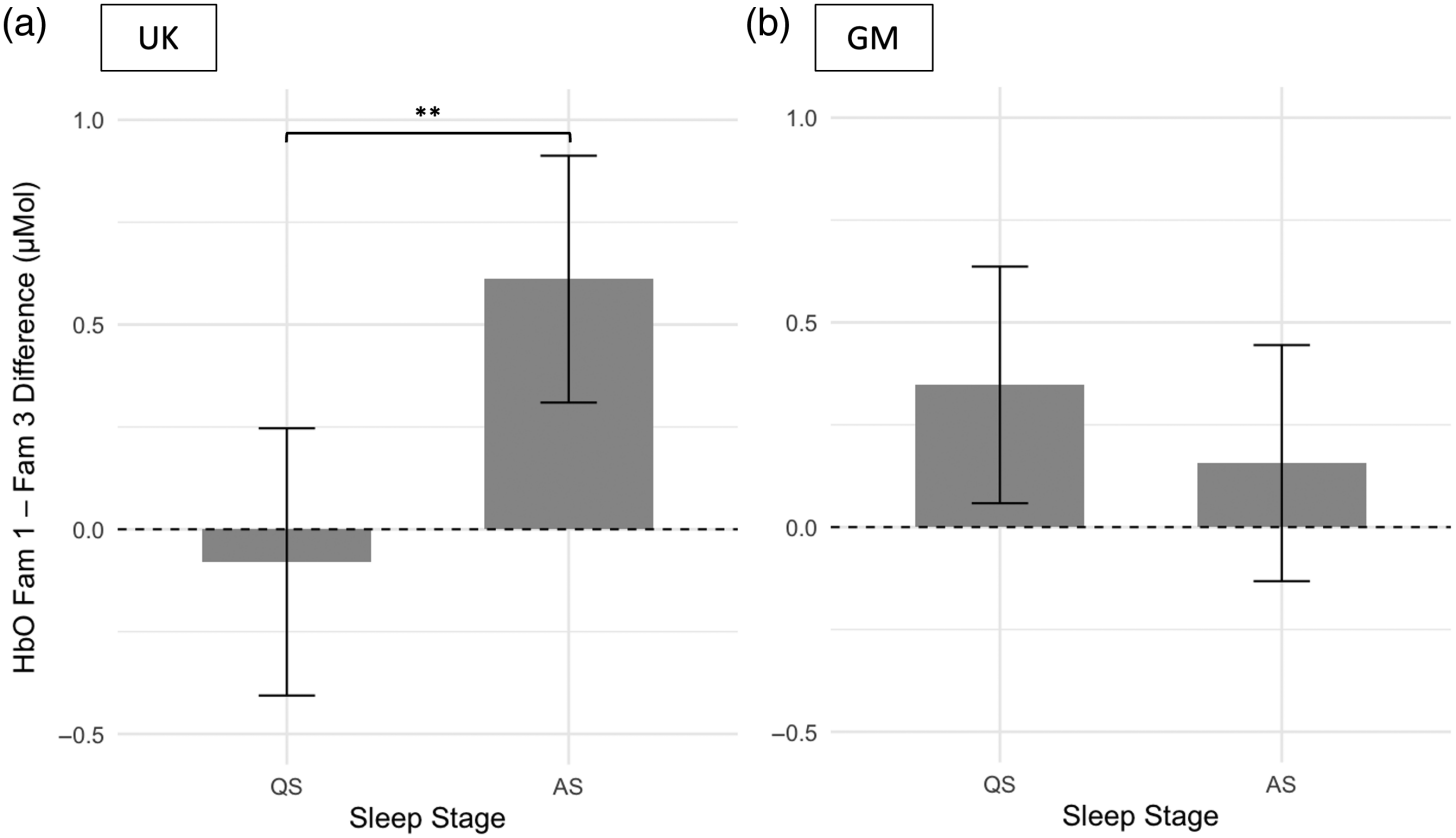
Bar plot showing the mean difference (Fam1 to Fam3) in HbO concentration for quiet (QS) and active (AS) sleep groups in the UK (a) and GM (b) cohorts. Error bars indicate standard errors. Asterisks indicate significance levels: *p<0.05, **p<0.01, ***p<0.001.

In the UK cohort, a two-sample t-test revealed a significant difference in habituation strength (Fam1 to Fam3) across sleep stages, with greater habituation observed in infants in AS for HbO (QS M=−0.08  μMol, SD=0.61; AS M=0.61  μMol, SD=0.45, t(25)=−3.19, p=0.004, Cohen’s d=−1.3 (large)] (Fig. 5) and HbR (QS M=−0.04  μMol, SD=0.20; AS M=−0.31  μMol, SD=0.26, t(27)=3.06, p=0.005, Cohen’s d=1.14 (large)] (Fig. S4 in the Supplementary Material). Permutation-based p-values and bootstrap confidence intervals for Cohen’s d are reported in Table S4 in the Supplementary Material and led to the same conclusion.

In the GM cohort, there were no significant differences in habituation values between sleep stages for HbO [(QS M=0.35  μMol, SD=0.70; AS M=0.16  μMol, SD=0.62, t(43)=0.96, p=0.34, Cohen’s d=0.29 (small)] (Fig. 5) or HbR [(QS M=−0.11  μMol, SD=0.22; AS M=−0.06  μMol, SD=0.19, t(43)=−0.94, p=0.39, Cohen’s d=−0.26 (small)] (Fig. S4 in the Supplementary Material; see also Table S4 in the Supplementary Material for full test statistics, permutation p-values, and bootstrap confidence intervals).

### 3.4 Results of the Channel-Wise Analyses

#### 3.4.1 Sleep-stage-specific spatial distribution of significant response to the social selectivity paradigm

**Fig. 6:**
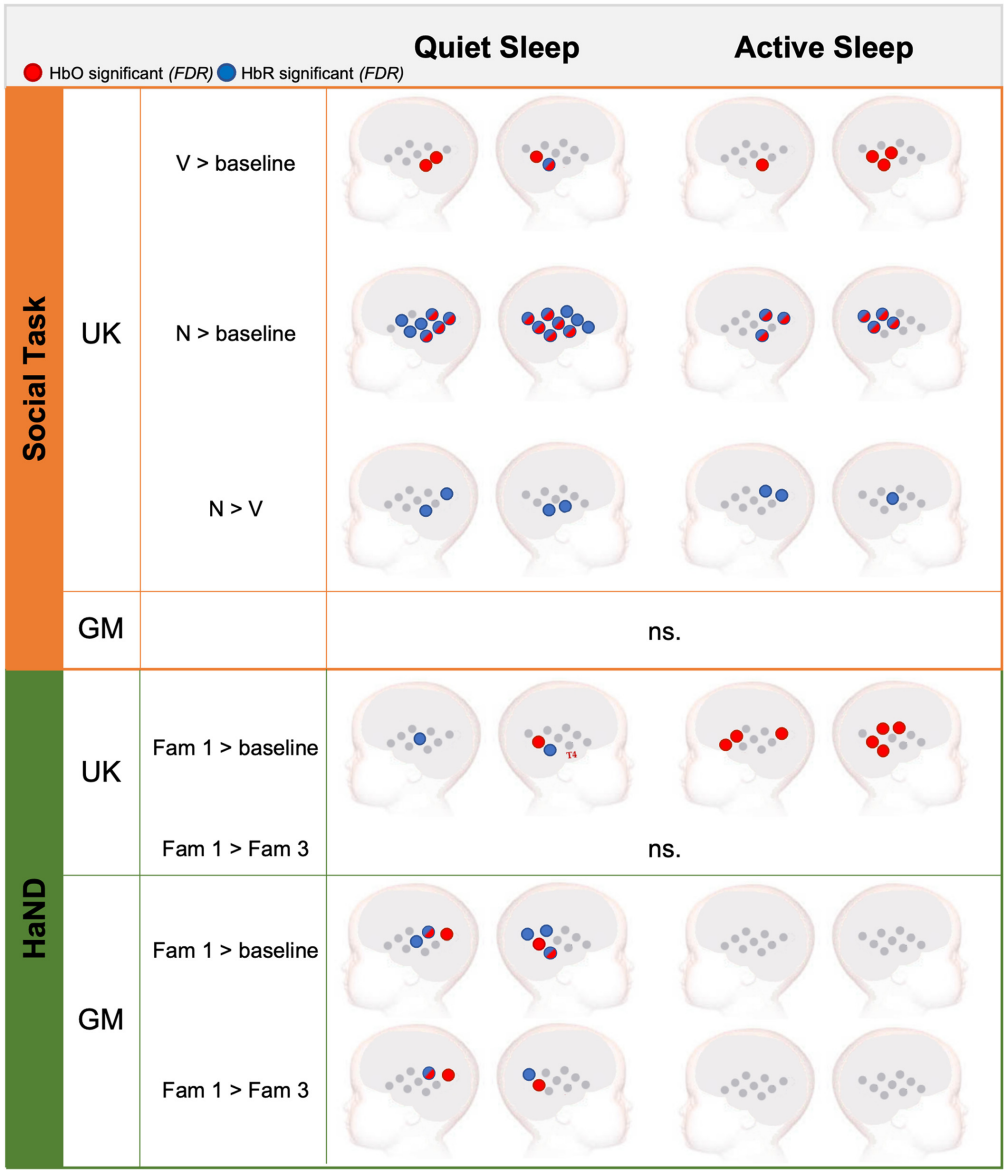
Results of the channel-wise analysis of hemodynamic activation for the social selectivity and HaND paradigms, showing only channels with significant effects after FDR correction (condition versus baseline: one-sample t-test, two-tailed, FDR-corrected, p adj<0.05; condition contrast: paired-sample t-test, two-tailed, FDR-corrected, p adj<0.05). Channels with significant HbO activation are shown in red, channels with significant HbR activation in blue, and channels significant for both in half-red/half-blue for the UK and GM infant cohorts. ns, not significant.

First, we determined the significance of the auditory response to V and N conditions separately in the AS and QS groups (one-sample t-test, two-tailed, FDR-corrected for the entire set of channels, only channels with FDR-adjusted p-values, p adj<0.05, are reported) (Fig. 6). In the UK cohort, in the QS group, significant activation was found in 4 out of 18 HbO channels (≈22%) and 1 HbR channel (≈6%) to V condition; activation to N condition was found in 10 HbO channels (≈56%) and 16 HbR channels (≈89%). In the AS group, significant activation to V condition was found in four HbO channels (≈22%) and zero HbR channels; activation to N condition was found in seven HbO channels (≈39%) and seven HbR channels (≈39%). A significant selectivity toward N > V condition (paired-sample t-test, two-tailed, FDR-corrected for the set of channels significant against baseline, p adj<0.05) was detected in four HbR channels (≈22%) in QS (5, 9, 17, and 18) and three HbR channels (≈17%) in AS (4, 5, and 16) but not in any HbO channels. There were no channels with significant V > N selectivity. The corresponding channel-wise effect sizes ranged from medium to large (Cohen’s d>0.57). See Table S5 in the Supplementary Material for full statistics, permutation-based p-values, and bootstrap confidence intervals for Cohen’s d. In the GM cohort, no channels showed significant HbO or HbR activation to either vocal or non-vocal condition; hence, N > V selectivity was not investigated in our GM subsample of infants.

#### 3.4.2 Sleep-stage-specific spatial distribution of significant response to the HaND paradigm

First, similarly to the social selectivity paradigm, we conducted channel-by-channel analysis of hemodynamic activation to Fam1 in AS and QS (one-sample t-test, two-tailed, FDR-corrected for the entire set of channels, p adj<0.05) (Fig. 6). In the UK cohort, infants in QS showed activation in 1 out of 18 HbO channels (≈6%) and 2 HbR channels (≈11%); infants in AS showed activation in seven HbO channels (≈39%) and zero HbR channels [the corresponding channel-wise effect sizes were all large (Cohen’s d>0.82); see Table S6 in the Supplementary Material for full statistics, permutation-based p-values, and bootstrap confidence intervals for Cohen’s d]. In the GM cohort, infants in QS showed activation in four HbO channels (≈22%) and five HbR channels (≈28%) [the corresponding channel-wise effect sizes ranged from medium to large (Cohen’s d>0.58); see Table S6 in the Supplementary Material for details]. Infants in AS had no significant activation in HbO or HbR channels. Next, channels with significant activation to Fam1 were tested for habituation (Fam1 > Fam3) or dishabituation (Fam3 > Fam1) separately for each sleep stage group (paired-sample t-test, two-tailed, FDR-corrected for the set of channels significant against baseline, p adj<0.05). In the UK cohort, we found that neither response was present in AS or QS. For the GM cohort, a significant habituation response was found in three HbO channels (≈17%) (channels 4, 5, and 15) and two HbR channels (≈11%) (channels 4 and 14) in the QS group [Cohen’s d values > 0.51 (medium-to-large); see Table S6 in the Supplementary Material for full statistics, permutation-based p-values, and bootstrap confidence intervals for Cohen’s d]. No channels with significant habituation or dishabituation response were identified in the AS group.

The results of the channel-by-channel analysis of hemodynamic activation to novel trials in AS and QS (one-sample t-test, two-tailed, and FDR-corrected for the entire set of channels) identified no HbO or HbR channels with significant activation in either cohort. Therefore, we did not proceed with the analysis of sleep stage effect on the presence of novelty response (novel > Fam3).

## 4 Discussion

This study examined the potential influence of sleep stage on infant brain hemodynamic responses across two auditory paradigms in UK and Gambian cohorts, using fNIRS. By leveraging both region-based and channel-wise analyses, we found that the effect of sleep stage on cortical processing was nuanced, depending on paradigm type and cohort. The results showed limited evidence of sleep stage effect on the response to the social paradigm, with only one outcome measure showing a significant sleep stage difference in one cohort. In contrast, the habituation paradigm showed clearer sleep stage effects on both the magnitude and habituation of responses, and these effects differed between the UK and Gambian cohorts. Across both tasks, channel-wise analyses highlighted differences in the spatial distribution of activation by sleep stage. These results suggest that sleep stage may modulate early auditory processing in task- and population-specific ways, underscoring the need to understand its effect in infant neuroimaging research.

### 4.1 Social Selectivity Paradigm

The analyses based on regions of interest defined in the full infant cohort,^55^ where a larger sample established robust paradigm-selective responses, allowed us to examine sleep stage effects on response amplitude and selectivity in regions with confirmed non-vocal > vocal responses. Overall, the amplitude of HbO responses to the non-vocal and vocal conditions did not differ across sleep stages in either cohort, and the strength of non-vocal selectivity (N > V) within the ROI identified by Greenhalgh et al.^55^ was not modulated by sleep stage for either HbO or HbR. The ROI-based analyses revealed a significant sleep stage effect on the HbR response in the UK cohort only, with infants in quiet sleep exhibiting a more negative (larger magnitude) response to the non-vocal condition than infants in active sleep. Taken together, these findings broadly align with previous fNIRS work reporting no sleep stage differences in response amplitude to speech and music stimuli in neonates^36^ and EEG studies showing no sleep stage differences in auditory change detection.^22^^–^^24^ The isolated HbR difference for the non-vocal condition in the UK cohort should be interpreted with caution and confirmed in larger samples before drawing firm conclusions about sleep stage effects on the processing of non-social auditory stimuli.

Channel-wise analyses using the subsample of infants with available sleep stage coding allowed us to examine the possible effect of sleep stage on the location of the response to social selectivity and habituation paradigms. In the UK cohort, infants in active and quiet sleep showed significant auditory response to both vocal and non-vocal stimuli in the medial and posterior temporal channels, with no apparent lateralization. The response to both types of stimuli was more widespread in quiet compared with active sleep with approximately double the number of active channels. Infants in both sleep stage groups discriminated between vocal and non-vocal stimuli with a significant selectivity for non-vocal stimuli, albeit only in the HbR signal. Despite wider activation in quiet sleep to both conditions, the number of channels with significant non-vocal preference was similar across sleep stage groups. The finding of non-vocal preference is in line with the results obtained using the full cohort of infants^55^ and with previous studies on this age group.^57^^,^^70^^,^^71^

In our subsample of the Gambian cohort, no channels showed significant activation to either condition relative to baseline; therefore, condition contrasts were not investigated. This partly contradicts the results obtained using the full cohort of infants where significant activation was found for both conditions. However, stimulus selectivity was not present in the full cohort. Hence, although the reduced sample used in this analysis could account for the inconsistent results, the investigation of condition contrasts in this paradigm at the group level would still not be possible even with a full sample. Interestingly, the full cohort paper also found a high level of inter-individual variability in social selectivity with some infants showing N > V and some V > N selectivity across both cohorts. Future work should explore this further as N over V discrimination was also associated with FC maturation patterns.^55^

In summary, our ROI-based and channel-wise analyses suggest that infants in both active and quiet sleep stages respond robustly to auditory stimuli and are equally able to discriminate between vocal and non-vocal sounds. In regions showing robust social selectivity at the group level, sleep stage was found to have little impact on response amplitude or selectivity, aside from a tentative HbR effect that warrants replication. Sleep stage did not affect spatial distribution or direction of stimulus selectivity. Importantly, although it is well established that non-vocal selectivity in early infancy typically shifts toward vocal preference with development, our results show that at 1 month of age, infants consistently exhibit non-vocal preference regardless of sleep stage. This finding is a valuable contribution to the field because it suggests that core auditory discrimination processes and early perceptual biases are preserved across sleep stages in two different populations of infants. Furthermore, by showing that sleep stage does not confound early markers of auditory selectivity, this study helps refine methodological approaches in infant neuroimaging and strengthens developmental models of early auditory social processing.

### 4.2 Habituation and Novelty Detection

Results based on the ROI defined by Greenhalgh et al.^55^ showed a higher average amplitude of the hemodynamic response to the Fam1 epoch in active sleep compared with quiet sleep in the UK cohort. Infants in active sleep also showed significantly stronger habituation, with an average HbO concentration decrease of 0.61  μMol between the first and last familiarization epochs. In contrast, infants who remained in quiet sleep showed a mean difference of −0.08  μMol between Fam1 and Fam3. This may reflect either absent habituation or rapid habituation occurring within Fam1 (trials 1 to 5), such that the block average already incorporates diminished responses. The possibility of rapid habituation in quiet sleep is consistent with our finding of significantly higher amplitude of response in Fam1 in active sleep versus quiet sleep infants.

Channel-wise analysis further supported greater response to the HaND stimuli in active sleep in the UK cohort, with more channels showing significant activation to Fam1 compared with quiet sleep. In active sleep, significant responses were found in bilateral frontal and posterior temporal regions, whereas in quiet sleep, activation was limited to posterior temporal regions. However, in the active sleep group, the activation was observed only in the HbO signal, whereas in quiet sleep, both HbO and HbR signals showed significant responses. In contrast to ROI-based analysis, no significant habituation patterns (channel-level differences between Fam1 and Fam3 epochs) were found in either sleep stage group. This contrasts with findings from the full UK cohort, where habituation effects were evident, suggesting that reduced power in our subsample may underlie the lack of significant habituation.

These findings align with Kotilahti et al.,^35^ who reported a stronger hemodynamic response to sinusoidal tones in active sleep compared with quiet sleep. However, a later study using more complex auditory stimuli (speech and music) found no effect of sleep stage on the strength of the response to either stimulus.^36^ Importantly, however, both studies involved newborns considerably younger than the participants in the present study. Hunter et al.^30^ found poor auditory sensory gating (S2/S1 ratio ≈1.0) during NREM/quiet sleep using EEG among 15-week-old infants, which could reflect either absent inhibition or rapid early habituation masked by averaging across 72 to 82 trials. However, their finding was stable regardless of the number of trials averaged, favoring absent habituation/gating. Given the differences in imaging modality (EEG versus fNIRS) and paradigm (sensory gating versus habituation), rapid early habituation within Fam1 remains plausible in our fNIRS data and warrants fine-grained trial-level analysis.

In the Gambian cohort, ROI-based analysis revealed no differences in average response amplitude to Fam1 across sleep stages. The strength of habituation was also not affected by sleep stage. However, according to the results of the channel-wise analysis, channels with significant activation to Fam1, as well as significant habituation response (Fam1 > Fam3), were found only in the quiet sleep group, whereas the response in active sleep did not differ from baseline in any of the channels. This contrasts with the results of the UK cohort and prior findings of stronger habituation in active sleep.^29^^,^^30^ The discrepancy may stem from the smaller sample size, higher inter-individual variability, and slightly lower data quality in the active sleep group in the Gambian sample. Alternatively, activation in quiet but not active sleep may reflect a cohort-specific hemodynamic response profile.

The presence of a sleep stage effect on the amplitude of the response to the habituation paradigm, but not to the social selectivity paradigm, may be partly explained by the differences in stimulus characteristics. The habituation paradigm used a relatively long, continuous 8-s spoken sentence, which likely engages sustained auditory and linguistic processing and may thus be more sensitive to cortical state fluctuations across sleep stages. In contrast, the social selectivity paradigm consisted of short, non-speech vocalizations (e.g., laughing, crying, and yawning) and various environmental sounds (toys, rattles, and bells), which are acoustically diverse and likely elicit hemodynamic responses that are more directly driven by the sensory properties of the stimuli—that is, processed in a more stimulus-dependent, bottom-up manner, less reliant on the modulatory influence of sleep stage. Furthermore, spoken sentences have exaggerated prosody and pitch contours designed to capture infant attention, possibly amplifying the differences between active and quiet sleep due to the differences in cortical excitability or sensory gating across sleep stages. Together, these differences in stimulus duration, complexity, and linguistic content may explain why sleep stage modulated responses in the habituation paradigm but not in the social selectivity paradigm.

The differences between our results and previous studies could be due to several methodological and developmental factors. First, our study included slightly older infants than some of the earlier work, which may have influenced how sleep stage modulates hemodynamic responses. As active and quiet sleep mature into REM and NREM sleep, respectively, these stages become more differentiated and structured, with distinct physiological and neural profiles. For example, sleep spindles are absent at birth, but develop rapidly within the first months of life, marking a transition from quiet sleep to NREM sleep. This changing sleep architecture means that the functional significance of sleep stages and their influence on sensory processing may vary substantially with age. Second, differences in stimulus type could also contribute to differences in findings, although our results indicate that this relationship is not straightforward. In our study, the more complex, speech-like stimuli of the habituation paradigm showed sleep stage differences in response amplitude, whereas the shorter non-speech sounds of the social selectivity paradigm did not, yet quiet sleep still recruited more channels overall in the latter. This indicates that the influence of sleep stages may not simply track stimulus complexity but rather reflect interactions among stimulus features, the developmental stage, and the neural networks engaged. Third, variations in sample size and resulting statistical power likely contribute, especially as our subsample stratified by sleep stage was considerably smaller than our full cohort analyses, potentially reducing sensitivity to detect habituation or condition contrasts. Finally, inconsistencies can also arise due to the differences in the neuroimaging modalities themselves; although fNIRS measures slow hemodynamic changes, MEG and EEG capture rapid electrophysiological dynamics, which may be differentially sensitive to sleep stage. Together, these factors highlight the multifaceted nature of sleep stage effects on early auditory processing and underscore the need for larger studies with different populations of infants to fully disentangle how sleep modulates infant brain responses.

## 5 Strengths and Limitations

One limitation of this study is the observed variation in data quality by sleep stage, which may have influenced the precision of our findings. Specifically, data quality and the number of valid trials were generally lower during active sleep compared with quiet sleep across both cohorts, with the exception of the number of vocal trials in the UK cohort, which did not differ significantly by sleep stage. Nevertheless, it is important to highlight that despite these differences, the proportion of clean data remained consistently high (above 95% across all conditions), indicating that although data acquisition may pose more challenges during active sleep, the quality was still sufficient to ensure the reliability of our analyses. Another limitation relates to sample size, which was reduced because some video recordings did not provide sufficient visibility of key behaviors and respiration cues (e.g., due to infant wrapping or positioning). This issue is common in naturalistic infant research, where it is challenging to standardize infant positioning and camera angles. Although adequate for detecting general patterns, our stratified subsample did not allow for more sensitive analyses such as TFCE, which were feasible in the full cohort. As a result, we were unable to examine whether the time window of the peak hemodynamic response differed by sleep stage—a factor that could be relevant, because differences in response latency might underlie some of the observed variability among stages. Finally, our reliance on behavioral sleep stage classification, based on the Brazelton Neonatal Behavioral Assessment Scale^65^ and the Anders Manual,^13^ represents a limitation. Although this method is practical and widely used in infant research, it lacks the precision of gold-standard multimodal approaches that combine EEG, ECG, respiration, and behavioral scoring for more accurate sleep stage coding.

These challenges highlight the value of developing automated methods for sleep stage classification based solely on neural, hemodynamic, or cardiac signals. Such approaches would not only improve the accuracy and objectivity of sleep staging but would also significantly reduce the need for time-intensive behavioral coding and specialized training, thereby enhancing the scalability of sleep-related research in infancy.

## 6 Conclusion

Our study offers several unique contributions to the field of developmental cognitive neuroscience and sleep research. First, our study stands out by investigating the effects of sleep stage in two populations of infants, which strengthens our findings by allowing us to examine the consistency of effects across different samples. Furthermore, we investigated sleep stage effects across two distinct auditory paradigms, commonly presented to young infants during sleep—social selectivity and habituation and novelty detection. Lastly, we employed two complementary analytical approaches: one guided by regions of interest defined from hemodynamic responses in the full cohort to maximize signal-to-noise ratio and another channel-wise approach that allowed us to explore potential sleep stage effects on the spatial distribution and presence of brain activation.

In summary, this analysis reveals that sleep stage modulates hemodynamic responses to repeated auditory stimuli in 1-month-old infants, with different patterns emerging across UK and Gambian cohorts. For the social selectivity paradigm, infants in both cohorts showed robust responses to vocal and non-vocal stimuli and non-vocal selectivity. Sleep stage did not affect HbO response amplitude or N – V selectivity in either cohort, although a sleep stage effect emerged in HbR in the UK cohort, with quiet sleep infants showing a stronger response to the non-vocal condition than active sleep infants. In the habituation paradigm, UK infants in active sleep exhibited a higher initial HbO response to the familiar stimulus and stronger habituation across repetitions in both HbO and HbR than quiet sleep infants. In the Gambian cohort, channel-wise effects were restricted to quiet sleep.

The results highlight the nuanced role of sleep stage in shaping hemodynamic responses during early infancy while also showing that patterns may differ across paradigms and populations. These findings underscore the importance of accounting for sleep stage in infant neuroimaging research and demonstrate the value of examining developmental processes across diverse settings. Future work with larger samples and targeted designs will be necessary to determine how population-specific factors and sleep architecture interact to influence early sensory and cognitive development.

## Supplementary Material

10.1117/1.NPh.13.S1. S13013.s01

## Data Availability

As per the BRIGHT Protocol paper,^53^ we provide the following information. We recognize the importance of maximizing outputs from the data collected in the BRIGHT project, both by serving the participants and communities that have agreed to partake in this research and the wider scientific community by providing access to the collected data for further analysis. Access to any data collected during or generated by the BRIGHT project is fully audited and, to ensure data security, is overseen by the data management team in the UK and the Gambia. Although data sharing is critically important to maximizing the benefit of research, we must also consider the need to protect the confidentiality of this sensitive group (particularly the infants within the mother–infant dyads, who as minors do not consent for themselves). Furthermore, to generate maximum value from this dataset, we must link data points together (i.e., NIRS/EEG data with outcome data or contextual factor data). Due to the nature of the data being collected (i.e., collected from a specific geographical location, longitudinal dataset of several data points), the majority of the data cannot be fully de-identified under the guidance included in the European General Data Protection Regulation (GDPR). The data used to support this study are stored in the Brain Imaging for Global Health Data Repository. The conditions of our ethics approval do not allow public archiving of pseudonymized study data. The data cannot be fully anonymized due to the nature of combined sources of information, such as neuroimaging, sociodemographic, geographic, and health measures, making it possible to attribute data to specific individuals, and hence, falling under personal information, the release of which would not be compliant with GDPR guidelines unless additional participant consent forms are completed. Our data sharing procedures were created in consultation with stakeholders and external consultation.^72^ Collaborations are encouraged, and projects are evaluated primarily on their consistency with the ethical principles and aims of the project that the families signed up to when partaking in this study. All planned analyses (both internal to the BRIGHT team and external) are pre-specified either on an internal database monitored by our management committee or via web-based pre-registration platforms. These procedures continue to be evaluated annually and updated to optimize the BRIGHT Project’s value to the scientific community and public priorities. To access the data, interested readers should contact the BRIGHT coordinator on our website (https://www.globalfnirs.org/contact/) or via https://github.com/globalfnirs. Access will be granted to named individuals following ethical procedures governing the reuse of sensitive data. Specifically, requestors must pre-register their proposal, and clearly explain the purpose of the analysis to ensure that the purpose and nature of the research is consistent with that to which participating families originally consented. In addition, requestors must complete and sign a data sharing agreement to ensure data are stored securely. Approved projects would need to adhere to the BRIGHT project’s policies on ethics, data sharing, authorship, and publication.
