# Supplementary material for: Effect of sleep stage on patterns of fNIRS hemodynamic response to auditory paradigms in 1-month-old Gambian and UK infants
Source: Neurophotonics. 2026 May 25;13(Suppl 1):S13013. doi: 10.1117/1.NPh.13.S1.S13013 (PMC13209660; doi:10.1117/1.NPh.13.S1.S13013)
Supplement: Supplementary file 1 [file NPh_013_S13013_SD001.pdf]

1 Supplemental Materials

1.1 *Sleep stages classification criteria*

Table S1 provides an overview of the criteria used for sleep stage classification and their associated weighting. Each criterion's percentage reflects its relative contribution to determining the sleep stage. For instance, 'Eyes closed/ Eyes Open' and 'Rapid Eye Movements (REM) versus Non-REM (NREM)' have the highest weighting at 25%, indicating their significant role in classification between the two stages. If any of the criteria can not be coded due to obscured visibility, the weighting of the obscured criterion is deducted from the total score of the 15-second coding epoch.

Table 1 Overview of the Sleep Classification Criteria and their Corresponding Level of Confidence.

Criterium	Weighting (%)
Eyes closed versus Eyes Open	25
REM versus NREM	25
Regular versus Irregular breathing	15
Jerky versus Smooth movement	15
Sucking	5
Facial Grimace	5
Vocalization	5
Startles	5

1.2 *Retention of participants based on fNIRS quality control*

Figure S1 shows participant retention per paradigm based on fNIRS data quality in the two cohorts. The data used in the current analysis was pre-processed as part of a separate investigation using the full dataset (Greenhalgh et al., 2025). In the Gambian (GM) cohort, of the total number of participants eligible at one month (N = 204), 136 had valid fNIRS data for at least the habituation part (Fam1, Fam2, Fam3) of the Habituation and Novelty Detection (HaND) paradigm and 148 had valid fNIRS data for the social selectivity paradigm. In the UK cohort, of the total number of participants eligible at one month (N = 61), 46 had valid fNIRS data for the social selectivity

paradigm and 38 for the HaND paradigm. Data collected as part of the functional connectivity (FC) paradigm was analysed as part of the separate full cohort investigation, but due to the small number of participants with valid FC and sleep stage coding, the effect of sleep stages on FC patterns was not investigated as part of the current work.

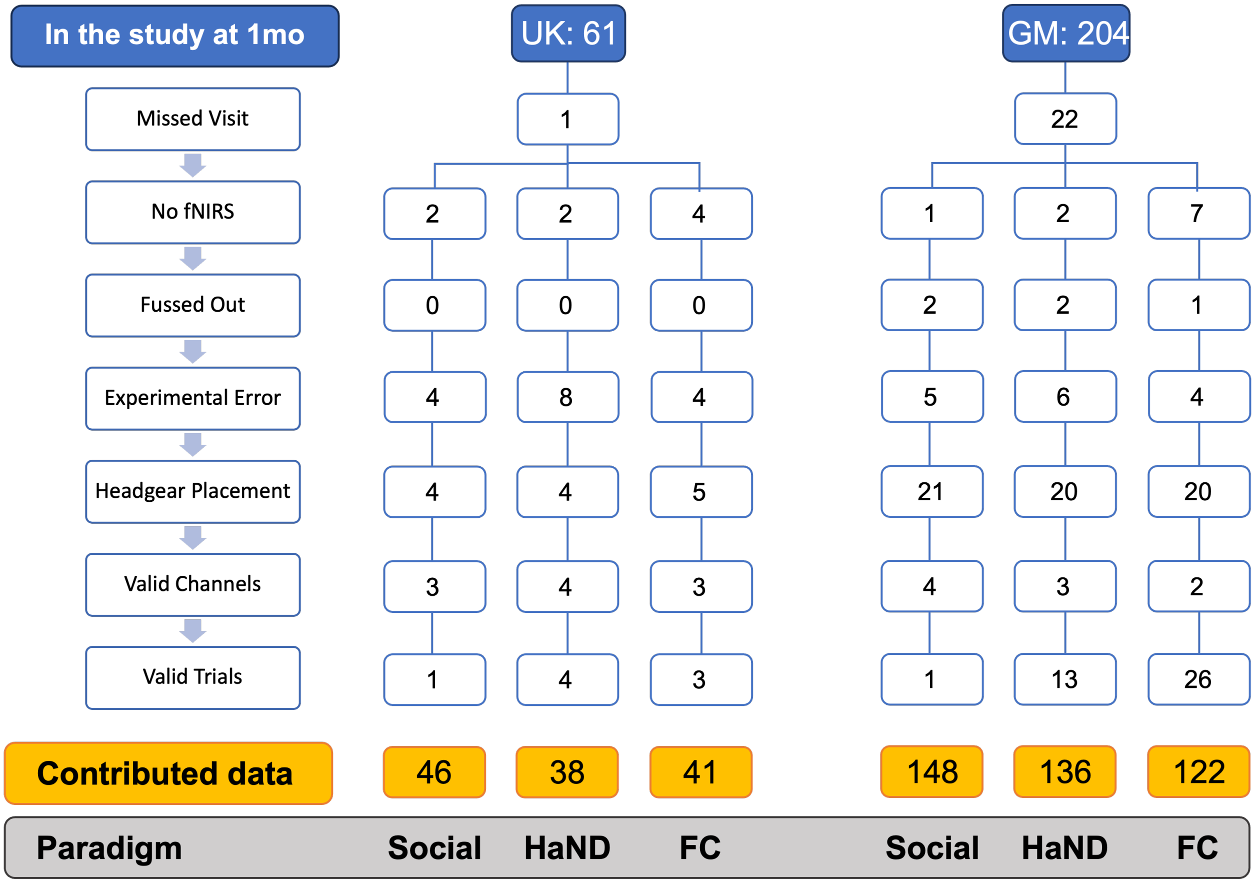

Fig 1 Reasons for exclusion of participants from the fNIRS analysis from the United Kingdom (UK) and Gambian (GM) cohorts across the three paradigms: vocal/non-vocal selectivity task (Social), Habituation and Novelty Detection (HaND) and functional connectivity (FC); The reasons for exclusion were: Missed Visit - participant did not attend the one-month session; No fNIRS - participant attended the one month session, but fNIRS data was not collected at all or for specific task; Fussed Out - infant woke up and cried during scanning so the recording had to be stopped; Experimental Error - collected data is invalid due to missing photos of the headband placement, missing event markers or technical issues; Headgear Placement - collected data is invalid due to wrong placement of the fNIRS headband; Valid Channels - collected data is invalid due to too few valid channels; Valid Trials - collected data is invalid due to too few valid trials.

Table 2 Group differences in hemodynamic responses to vocal (V) and non-vocal (N) conditions of the social selectivity paradigm by cohort, chromophore, and sleep stage

Cohort	Measure	Condition	*n* (QS)	*n* (AS)	*Mean* QS	*SD* QS	*Mean* AS	*SD* AS	*t*	*df*	*p*	Permutation *p*	Bootstrap *Cohen's d* (95% *CI*)
UK	HbO	N	17	21	0.50	0.43	0.59	0.80	-0.42	32	0.675	0.682	-0.13 [-0.74, 0.51]
		V	18	21	0.37	0.41	0.43	0.49	-0.40	37	0.690	0.688	-0.13 [-0.74, 0.54]
	HbR	N	18	17	-0.22	0.14	-0.09	0.15	-2.75	32	0.010	0.006	-0.93 [-1.61, -0.36]
		V	18	20	-0.14	0.15	-0.07	0.18	-1.41	36	0.168	0.165	-0.45 [-1.17, 0.15]
GM	HbO	N	20	25	0.10	0.38	0.17	0.47	-0.55	43	0.584	0.572	-0.16 [-0.77, 0.4]
		V	20	24	0.28	0.46	0.15	0.36	1.08	36	0.286	0.281	0.34 [-0.28, 1.04]
	HbR	N	19	25	-0.03	0.07	-0.04	0.10	0.09	42	0.932	0.934	0.03 [-0.59, 0.61]
		V	19	25	-0.02	0.07	-0.04	0.10	0.81	42	0.422	0.430	0.24 [-0.35, 0.82]

1.3 Sleep stage effects on the amplitude of significant responses

Table S2 presents the results of ROI-based analyses comparing the mean amplitude of significant oxyhemoglobin (HbO) and deoxyhemoglobin (HbR) responses to vocal (V) and non-vocal (N) conditions of the social selectivity paradigm between sleep-stage groups. In the UK cohort, infants in quiet sleep (QS) showed a significantly higher mean HbR response to the N condition than infants in active sleep (AS), whereas HbO responses to either condition did not differ significantly between sleep stages. In the GM cohort, no significant differences in mean HbO or HbR response amplitude were observed between sleep stages for either condition.

Table S3 presents ROI-based analyses comparing the mean amplitude of significant responses to the first five familiarization trials (Fam1) of the HaND paradigm between sleep-stage groups. In the UK cohort, infants in active sleep (AS) showed a higher-amplitude HbO response than infants in quiet sleep (QS), whereas HbR response amplitudes did not differ significantly between sleep-stage groups in either cohort (Figure S2).

Table 3 Group differences in hemodynamic responses to first five familiarization trials (Fam1) of the HaND paradigm by cohort, chromophore, and sleep stage

%

Cohort	Measure	*n* (QS)	*n* (AS)	*Mean* QS	*SD* QS	*Mean* AS	*SD* AS	*t*	*df*	*p*	Permutation *p*	Bootstrap *Cohen's d* (95% *CI*)
UK	HbO	16	13	0.15	0.37	0.62	0.51	-2.72	21	0.013	0.008	-1.05 [-1.97, -0.41]
	HbR	17	13	-0.13	0.13	-0.20	0.14	1.27	25	0.217	0.217	0.47 [-0.24, 1.34]
GM	HbO	25	24	0.19	0.41	0.22	0.56	-0.28	42	0.783	0.777	-0.08 [-0.68, 0.48]
	HbR	25	24	-0.09	0.12	-0.10	0.20	0.17	37	0.863	0.865	0.05 [-0.56, 0.61]

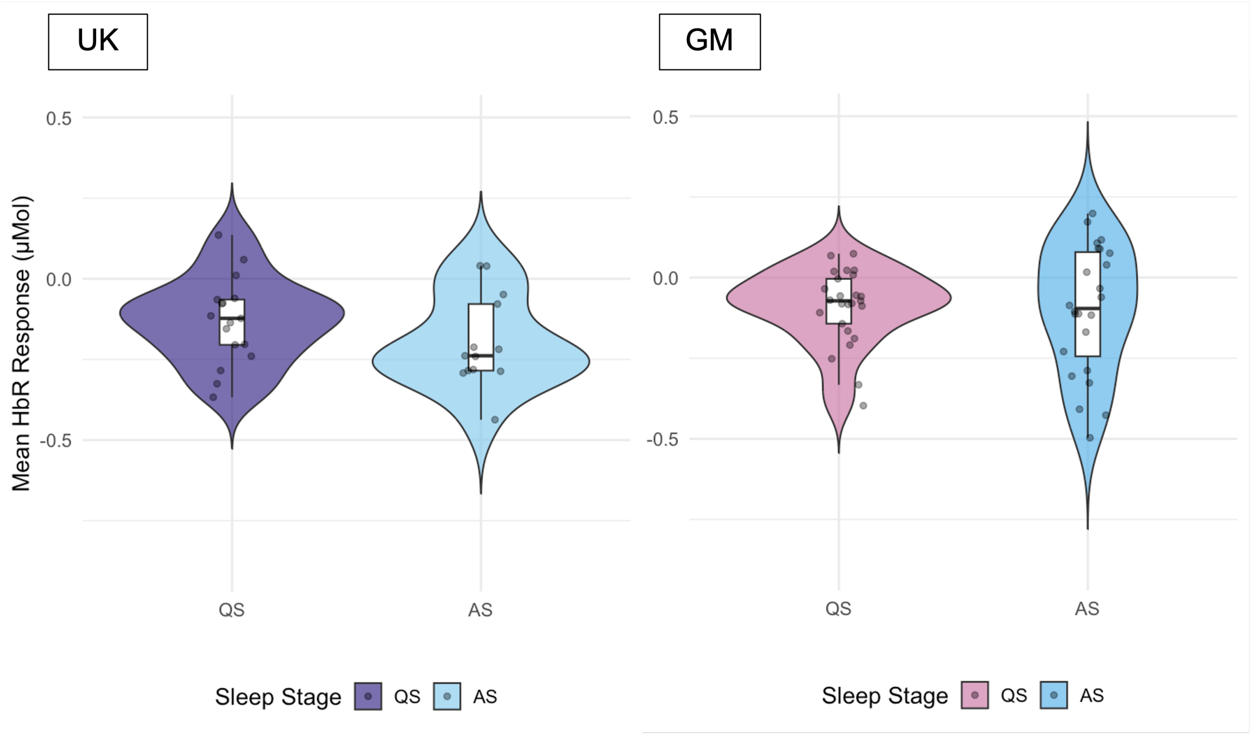

Fig 2 The distribution of mean deoxyhemoglobin (HbR) response amplitudes (μMol) to first five familiarization trials (Fam1) of the habituation and novelty detection (HaND) task across sleep states (QS = Quiet Sleep, AS = Active Sleep) in the UK (right) and Gambian (GM) (left) cohorts.

1.4 The effect of sleep stages on the strength of non-vocal selectivity

Figure S3 presents ROI-based analyses assessing whether sleep stage influenced the strength of non-vocal selectivity - that is, the extent to which infants responded more strongly to non-vocal than vocal stimuli of the social selectivity paradigm. In the UK cohort, significant N $>$ V selectivity was identified in the full cohort analysis (Greenhalgh et al., 2025). In the GM cohort, no regions showed either N $>$ V or V $>$ N selectivity. Therefore, the current ROI-based analyses of sleep stage effects on the strength of N $>$ V selectivity were restricted to the UK cohort. A two-sample, two-tailed t-test comparing average N–V condition contrast values between sleep stage groups revealed no significant differences for either HbO or HbR in the UK cohort.

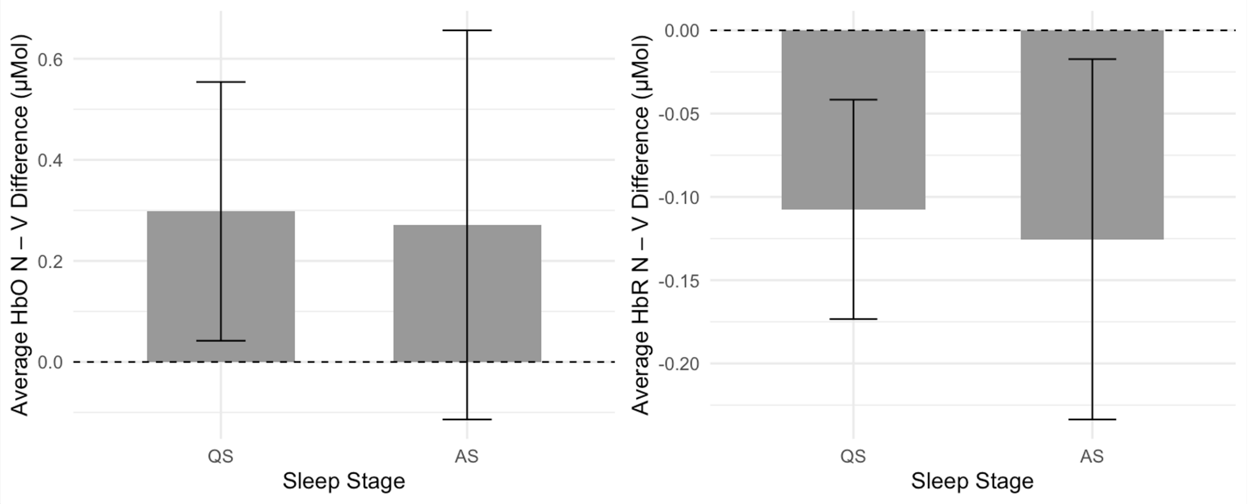

Fig 3 Mean difference in oxyhemoglobin (HbO) (left) and deoxyhemoglobin (HbR) (right) concentration between Non-vocal (N) and Vocal (V) trials during the social selectivity paradigm, shown separately for Quiet Sleep (QS) and Active Sleep (AS) for the UK cohort. Bars represent group means; error bars indicate 95% confidence intervals. For HbO, positive values reflect a greater HbO increase (i.e. stronger response) to N compared to V stimuli. For HbR, a negative value reflects a stronger response to Non-vocal compared to Vocal stimuli.

1.5 The effect of sleep stage on habituation strength

Table S4 and Figure S4 present ROI-based analyses assessing whether sleep stage influenced the strength of habituation - that is, the degree to which infants responded more strongly to the first five familiarization trials (Fam1) compared to the last five familiarization trials (Fam3) of the HaND paradigm. In the UK cohort, infants in AS showed greater habituation in their HbO and HbR response compared to QS infants. In the GM cohort, HbO or HbR response was not significantly different between the sleep stage groups.

Table 4 Habituation strength (Fam1–Fam3 difference) in hemodynamic responses by cohort, chromophore, and sleep stage (ROI-based analyses).

%

Cohort	Measure	*n* (QS)	*n* (AS)	*Mean* QS	*SD* QS	*Mean* AS	*SD* AS	*t*	*df*	*p*	Permutation *p*	Bootstrap *Cohen's d* (95% *CI*)
UK	HbO	16	11	-0.08	0.61	0.61	0.45	-3.19	25	0.004	0.002	-1.25 [-2.47, -0.52]
UK	HbR	16	13	-0.04	0.20	-0.31	0.26	3.06	27	0.005	0.007	1.14 [0.44, 2.17]
Gambia	HbO	25	20	0.35	0.70	0.16	0.62	0.96	43	0.343	0.332	0.29 [-0.32, 0.86]
Gambia	HbR	25	20	-0.11	0.22	-0.06	0.19	-0.87	43	0.387	0.365	-0.26 [-0.87, 0.31]

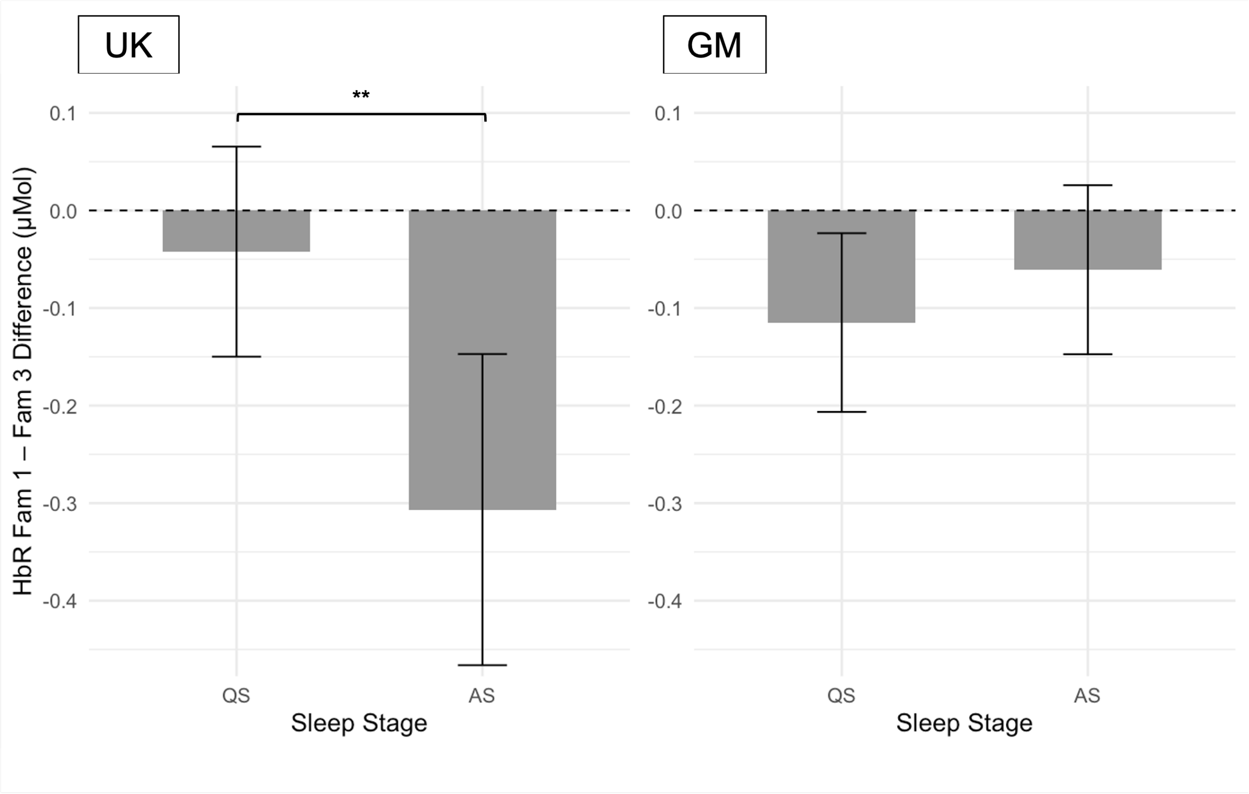

Fig 4 Mean difference (Fam1 - Fam3) in deoxyhemoglobin (HbR) concentration by sleep stage in the UK (right) and Gambian (GM) (left) cohort. Error bars indicate standard errors.

1.6 Sleep-stage specific spatial distribution of significant response to the social selectivity paradigm

Table S5 reports the full channel-wise statistics for the social paradigm. For each site, sleep stage, and channel, the table presents *t*-values, degrees of freedom, FDR-corrected *p*-values, permutation-based *p*-values, and Cohen's *d* with bootstrap 95% confidence intervals for (i) condition–baseline contrasts (N vs baseline and V vs baseline) and (ii) the condition contrast (N vs V). Only channels showing significant effects after FDR correction in at least one analysis are listed.

Table 5 The channel-by-channel two-tailed t-test analysis for the contrast between the conditions (Vocal and Non-Vocal) of the social selectivity paradigm and the baseline (silence), and for the contrast between the conditions (Non-Vocal > Vocal). For each contrast, results for significant increases in HbO and/or decreases in HbR concentration (FDR-corrected) are displayed.

		HbO (μMol)							*HbR (μMol)*						
		**Ch**	t	*p (FDR)*	*df*	Permutation *p*	Bootstrap *Cohen's d*	*Cohen's d95% CI*	**Ch**	*t*	*p (FDR)*	*df*	Permutation *p*	Bootstrap *Cohen's d*	*Cohen's d95% CI*
		**Non-vocal condition vs baseline**													
**UK**	**Quiet Sleep**	**4**	3.20	0.011	17	0.001	0.85	0.47, 1.46	**2**	-3.54	0.005	12	0.004	-1.06	-1.85,-0.48
		**5**	4.25	0.002	17	0.001	1.04	0.65, 1.56	**4**	-3.60	0.004	17	0.001	-0.92	-1.51, -0.47
		**6**	4.01	0.003	17	<0.001	0.99	0.60, 1.46	**5**	-4.26	0.001	17	0.001	-1.05	-1.56, -0.62
		**9**	4.60	0.002	17	<0.001	1.14	0.76, 1.64	**6**	-5.06	¡0.001	17	<0.001	-1.26	-2, -0.77
		**13**	3.67	0.006	17	0.002	0.90	0.47, 1.5	**7**	-3.45	0.005	17	0.004	-0.89	-1.54, -0.37
		**14**	4.57	0.002	16	<0.001	1.19	0.81, 1.74	**8**	-3.61	0.004	17	<0.001	-0.9	-1.35, -0.55
		**15**	4.65	0.002	17	<0.001	1.16	0.81, 1.62	**9**	-6.81	¡0.001	17	<0.001	-1.69	-2.5, -1.12
		**16**	2.95	0.016	17	0.011	0.76	0.24, 1.39	**10**	-2.29	0.046	12	0.044	-0.69	-1.36, -0.15
		**17**	3.27	0.010	17	0.001	0.81	0.50, 1.19	**11**	-2.30	0.046	12	0.038	-0.76	-1.93, -0.12
		**18**	3.58	0.006	17	0.003	0.89	0.47, 1.42	**12**	-3.54	0.005	12	0.003	-1.05	-1.71, -0.58
									**13**	-4.60	0.001	17	<0.001	-1.14	-1.78, -0.66
									**14**	-5.40	¡0.001	16	<0.001	-1.41	-2.15, -0.89
									**15**	-6.54	¡0.001	17	<0.001	-1.67	-2.46, -1.02
									**16**	-5.34	¡0.001	17	<0.001	-1.33	-1.95, -0.84
									**17**	-4.92	¡0.001	17	<0.001	-1.22	-1.98, -0.69
									**18**	-5.61	¡0.001	17	<0.001	-1.43	-2.22, -0.94
	**Active Sleep**	**4**	2.78	0.034	18	0.007	0.68	0.32, 1.05	**4**	-3.43	0.013	18	0.001	-0.83	-1.24, -0.51
		**5**	3.42	0.021	19	<0.001	0.82	0.50, 1.28	**5**	-3.78	0.008	19	<0.001	-0.88	-1.2, -0.6
		**9**	3.68	0.021	19	<0.001	0.88	0.56, 1.28	**9**	-3.77	0.008	19	<0.001	-0.91	-1.49, -0.47
		**13**	2.84	0.034	20	0.006	0.64	0.27, 1.05	**13**	-2.55	0.049	20	0.016	-0.58	-1.01, -0.19
		**14**	3.03	0.031	19	0.004	0.71	0.34, 1.12	**14**	-2.75	0.038	19	0.012	-0.64	-1.08, -0.24
		**15**	2.74	0.034	19	0.013	0.64	0.25, 1.07	**15**	-3.85	0.008	19	0.001	-0.92	-1.55, -0.41
		**16**	3.31	0.021	20	0.003	0.74	0.37, 1.15	**16**	-2.98	0.027	20	0.005	-0.67	-1.03, -0.37
		**Vocal condition vs baseline**													
	**Quiet Sleep**	**6**	4.06	0.009	17	0.001	0.99	0.58, 1.49	**18**	-4.12	0.013	17	<0.001	-1.01	-1.61, -0.55
		**9**	3.45	0.014	17	0.004	0.86	0.39, 1.48							
		**15**	3.64	0.012	17	0.002	0.94	0.41, 1.64							
		**18**	3.97	0.009	17	0.002	1.06	0.43, 1.98							
	**Active Sleep**	**9**	3.10	0.026	19	0.003	0.74	0.40, 1.15							
		**15**	3.10	0.026	19	0.005	0.72	0.34, 1.18							
		**16**	3.46	0.022	20	0.003	0.77	0.40, 1.24							
		**18**	3.86	0.019	19	0.001	0.90	0.48, 1.44							
		**Non-vocal ¿ Vocal Condition**													
	**Quiet Sleep**								**3**	-5.11	0.001	17	<0.001	-1.30	-2.15, -0.72
									**7**	-3.29	0.023	17	0.004	-0.81	-1.26, -0.42
									**11**	-3.99	0.008	17	<0.001	-1.08	-1.80, -0.71
									**12**	-3.16	0.024	16	0.005	-0.81	-1.26, -0.42
	**Active Sleep**								**1**	-3.69	0.012	18	0.001	-0.88	-1.24, -0.58
									**2**	-2.78	0.041	19	0.003	-0.64	-0.92, -0.37
									**7**	-2.54	0.046	20	0.015	-0.57	-0.98, -0.2

1.7 Sleep-stage specific spatial distribution of significant response to the Habituation and Novelty Detection (HaND) paradigm

Table S6 reports the full channel-wise statistics for the HaND paradigm. For each site, sleep stage, and channel, the table presents *t*-values, degrees of freedom, FDR-corrected *p*-values, permutation-based *p*-values, and Cohen's *d* with bootstrap 95% confidence intervals for (i) the first familiarisation epoch – baseline contrasts (Fam1 vs baseline) and (ii) the habituation acros epochs (Fam1 vs Fam3). Only channels showing significant effects after FDR correction in at least one analysis are listed.

Table 6 The channel-by-channel two-tailed t-test analysis for the contrast between the first familiarisation epoch (Fam1) and the baseline (silence), and for the contrast between the first and last familiarisation epoch (Fam3). For each contrast, results for significant increases in HbO and/or decreases in HbR concentration (FDR-corrected) are displayed.

		HbO (µMol)							*HbR (µMol)*						
		**Ch**	*t*	*p (FDR)*	*df*	Permutation *p*	Bootstrap *Cohen's d*	*Cohen's d* 95% *CI*	**Ch**	*t*	*p (FDR)*	*df*	Permutation *p*	Bootstrap *Cohen's d*	*Cohen's d* 95% *CI*
		**Fam1 vs baseline**													
	**Quiet Sleep**	**15**	5.81	0.001	15	< 0.001	1.58	0.86, 3.01	**7**	-3.27	0.043	16	0.005	-0.84	-1.50, -0.33
									**18**	-4.35	0.010	15	0.001	-1.15	-1.80, -0.66
	**Active Sleep**	**2**	3.36	0.034	14	0.007	0.97	0.32, 2.11							
		**3**	3.18	0.034	14	0.006	0.87	0.37, 1.56							
**UK**		**5**	3.09	0.034	12	0.007	0.98	0.42, 1.85							
		**10**	3.02	0.034	13	0.008	0.89	0.29, 1.88							
		**13**	2.77	0.039	14	0.017	0.82	0.22, 1.60							
		**15**	4.34	0.014	13	0.001	1.25	0.71, 2.08							
		**18**	2.95	0.034	13	0.014	0.87	0.30, 1.65							
		**Fam1 vs baseline**													
	**Quiet Sleep**	**4**	3.25	0.021	26	0.002	0.65	0.32, 1.01	**4**	-3.47	0.017	26	0.001	-0.68	-0.98, -0.40
		**5**	4.30	0.004	25	< 0.001	0.89	0.42, 1.51	**7**	-2.85	0.037	25	0.003	-0.58	-0.90, -0.27
		**15**	2.85	0.040	24	0.009	0.59	0.22, 1.00	**13**	-3.60	0.017	26	0.001	-0.73	-1.21, -0.30
		**18**	3.22	0.021	25	0.001	0.65	0.40, 0.94	**14**	-2.80	0.037	23	0.011	-0.59	-1.05, -0.20
**GM**									**18**	-3.13	0.026	25	0.003	-0.63	-0.95, -0.33
		**Fam1 vs Fam3**													
		**4**	3.47	0.008	24	0.002	0.73	0.30, 1.25	**4**	-3.12	0.021	24	0.004	-0.65	-1.10, -0.29
		**5**	2.42	0.045	23	0.015	0.52	0.15, 0.87	**14**	-2.91	0.021	21	0.008	-0.65	-1.15, -0.22
		**15**	2.26	0.045	22	0.029	0.51	0.07, 1.03							
